# High-Resolution Frequency-Domain Spectroscopic and Modeling Studies of Photosystem I (PSI), PSI Mutants and PSI Supercomplexes

**DOI:** 10.3390/ijms25073850

**Published:** 2024-03-29

**Authors:** Valter Zazubovich, Ryszard Jankowiak

**Affiliations:** 1 Department of Physics, Concordia University, Montreal, QC H4B 1R6, Canada; 2 Department of Chemistry, Kansas State University, Manhattan, KS 66506, USA; ryszard@ksu.edu

**Keywords:** Photosystem I, energy transfer, charge transfer, optical spectroscopy, spectral-hole burning, fluorescence

## Abstract

Photosystem I (PSI) is one of the two main pigment–protein complexes where the primary steps of oxygenic photosynthesis take place. This review describes low-temperature frequency-domain experiments (absorption, emission, circular dichroism, resonant and non-resonant hole-burned spectra) and modeling efforts reported for PSI in recent years. In particular, we focus on the spectral hole-burning studies, which are not as common in photosynthesis research as the time-domain spectroscopies. Experimental and modeling data obtained for trimeric cyanobacterial Photosystem I (PSI_3_), PSI_3_ mutants, and PSI_3_–IsiA_18_ supercomplexes are analyzed to provide a more comprehensive understanding of their excitonic structure and excitation energy transfer (EET) processes. Detailed information on the excitonic structure of photosynthetic complexes is essential to determine the structure–function relationship. We will focus on the so-called “red antenna states” of cyanobacterial PSI, as these states play an important role in photochemical processes and EET pathways. The high-resolution data and modeling studies presented here provide additional information on the energetics of the lowest energy states and their chlorophyll (Chl) compositions, as well as the EET pathways and how they are altered by mutations. We present evidence that the low-energy traps observed in PSI are excitonically coupled states with significant charge-transfer (CT) character. The analysis presented for various optical spectra of PSI_3_ and PSI_3_-IsiA_18_ supercomplexes allowed us to make inferences about EET from the IsiA_18_ ring to the PSI_3_ core and demonstrate that the number of entry points varies between sample preparations studied by different groups. In our most recent samples, there most likely are three entry points for EET from the IsiA_18_ ring per the PSI core monomer, with two of these entry points likely being located next to each other. Therefore, there are nine entry points from the IsiA_18_ ring to the PSI_3_ trimer. We anticipate that the data discussed below will stimulate further research in this area, providing even more insight into the structure-based models of these important cyanobacterial photosystems.

## 1. Introduction

Photosynthesis is a complex sequence of biophysical and biochemical processes directly or indirectly responsible for most of life on Earth. In plants, green algae and cyanobacteria, the first steps of this sequence, occur in large pigment–protein complexes (“photosynthetic complexes”, PC) located in the thylakoid membranes of the chloroplasts [1,2]. Photosystem I (PSI) is one of these large complexes. More details on how PSI fits into the bigger picture of oxygenic photosynthesis can be found in other articles of this special issue.

Optical methods focus on the interactions between pigment–protein complexes and light and they are therefore particularly suitable for exploring the very first, or primary, processes of photosynthesis, such as light harvesting, excitation energy transfer (EET) and primary charge separation. PCs are very complex biological systems containing dense networks of chlorophylls (Chls) and other pigments. They perform as either light-harvesting antennae or as reaction centers (RC) where primary photochemistry takes place [1,2,3]. High effectiveness of light-harvesting, EET, and electron transfer (ET) are achieved, as these processes occur on very fast, picosecond and sub-picosecond time scales [4,5,6,7,8,9,10,11,12,13,14,15,16,17,18,19,20,21,22,23,24]. Such fast processes can be explored with both time-domain and/or frequency-domain spectroscopy methods.

Widely used ultrafast spectroscopy techniques include pump–probe [4,5,6,7,8,9,10], two-dimensional electronic spectroscopy (2DES) [11,12,13,14,15,16,17,18,19,20,21], and photon echo [22,23,24,25,26,27,28,29]. Time domain experiments provide transient responses of the system (PSI in this case) induced by an external excitation, yielding detailed information on the EET between pigments absorbing at different wavelengths in a broad variety of photosynthetic complexes. For the subsequent discussion, it is important to realize that even though the structures of the PC are well-defined, some degree of inhomogeneity of the local environment is still preserved, resulting in the distributions of transition energies for pigments that are in supposedly identical local environments. It was proposed that different types of disorder can be distinguished each from other, and the bath correlation function can be obtained directly from various photon echo peak measurements. At cryogenic and room temperatures, 2DES reveals excitation energy relaxation and transport, as well as vibrational dynamics, in various molecular systems. The 2DES can also reveal excitation energy relaxation processes and many details about the fluctuating environment. By studying the spectral line shapes, 2DES can reveal the fluctuation spectral densities for different electronic states, the interstate correlation of static disorder (if present), and the timescales of spectral diffusion with high resolution [11,12,14,15]. Excellent general reviews of time-domain techniques can be found in [30,31]. The global and target analysis of the time-resolved spectra [32] is often used to extract information from the overwhelming amounts of data to describe very complex combinations of the processes above. On the other hand, the Fourier transform (FT) relationship between time and frequency results in a decrease of spectral resolution with the increase of time resolution. Spectral resolution is important in PC research, as the absorption of hundreds of (chemically identical, but with different local environment) pigments may be cramped into a relatively narrow spectral range. In addition, high-quality time-resolved spectroscopy systems are generally very expensive.

Frequency-domain techniques exploit the FT relationship above to obtain the characteristic times of EET and ET processes from the spectral line widths. They rely on tunable narrow-band lasers and conventional and/or FT spectrometers. Both time- and frequency-domain techniques are widely used in photosynthesis research and provide complementary data. Therefore, a comprehensive understanding of complex photosystems requires application of both classes of techniques. Since both classes cannot be adequately addressed in a short review, this work focuses only on low-temperature frequency-domain spectroscopies, including absorption, emission, and circular dichroism (CD), as well as site-selective high-resolution ones, which provide complementary information to the time-domain data. The high-resolution techniques described below include fluorescence line-narrowing (FLN) [33,34] and various types of high-resolution hole-burning (HB) spectroscopies [34,35,36,37,38,39,40,41], as well as a combination of FLN and HB, i.e., difference-FLN spectroscopy (∆FLN) [34,42,43]. These low-temperature techniques can overcome the effects of inhomogeneous broadening, providing, for example, information on inhomogeneous broadening, the single-site (molecule or excitonic state) line shape, mean phonon frequency, electron–phonon (el-ph) coupling strength, and phonon and vibrational spectral densities, i.e., *J_ph_*(*ω*) and *J_vib_*(*ω*), respectively [34,43,44,45]. These spectral densities are important for modeling and understanding EET processes. Another method of overcoming inhomogeneous broadening, also briefly addressed below, is single pigment–protein complex spectroscopy (SPCS). This technique allows us (for very low-concentration samples) to probe photosynthetic complexes one by one [46,47,48]. In this case, data supposedly matching that of ensemble measurements (if needed) can be assembled by binning information for multiple individual complexes.

To deduce the excitonic structure and dynamics of pigment–protein complexes, it is additionally essential to know (i) the pigments’ site-energies (transition energies in the absence of inter-pigment coupling); (ii) the interactions between pigments; and (iii) the dynamic modulation of the pigment energy (represented by the spectral density) induced by the environment. Proper values of site energies, phonon spectral densities, and el-ph coupling strengths are essential to understand the excitonic structure and EET dynamics, as well as EET and ET pathways in photosynthetic pigment–protein complexes. While spectral densities can be measured experimentally [43,49] and coupling matrix elements can be calculated relatively easily (assuming X-ray structures are known; several approaches are available [50,51,52,53]), the site-energies are typically extracted from simultaneous fits to various experimental data [54,55,56,57]. Quantum chemical approaches are also used [50,58,59], but calculated site energies exhibit some systematic errors and must be further optimized by various fitting algorithms [51,55,57,60,61].

Below, we present recent experimental data, as well as advances in the modeling of optical spectra of cyanobacterial PSI, focusing on low-temperature frequency-domain results. Although PSI is one of the best understood PCs, several issues are not yet fully resolved. For instance, PSI possess spectral bands that are lower in energy than the P700 primary (electron) donor. The exact structural/molecular origins of these “red states” (i.e., which of the ~100 Chls per cyanobacterial PSI monomer contribute to the “red states”) and their effects on EET kinetics are not fully understood. In this review, we discuss the nature of the low-energy states uncovered via various techniques and modeling studies over the years [3]. Knowing the excitonic structure of photosynthetic complexes is essential to understand structure–function relationship, as the red states are believed to play an important role in EET pathways.

We particularly focus on various spectral hole-burning studies [62,63,64,65,66,67,68,69,70,71,72], which are not as common and perhaps not as well appreciated as many of the time-resolved spectroscopies [4,5,6,7,8,9,10,11,12,13,14,15,16,17,18,19,20,21,22,23,24,25,26,27,28,29]. One can also see this review as a set of examples of what can be done with these methods in photosynthesis research. The continued improvement of frequency-domain spectroscopies should foster further use of these types of investigations in the future. The PCs discussed in detail below include wild-type (WT) trimeric cyanobacterial PSI_3_ complexes, PSI_3_ mutants, and PSI_3_–IsiA_18_ supercomplexes (grown under iron-deficient conditions) of *Synechocystis* PCC 6803. We discuss the high-resolution data that provide information on the energetics of the lowest energy states and their Chl compositions, as well as the mutation-altered EET pathways. We also address the similarities between the “red states” identified in *Synechocystis* PCC 6803 with those in *T. elongatus*, whose structure has been known for a longer time and which has been therefore explored in more detail. (The latter cyanobacterium is also known as *Synechococcus elongatus* and *Thermosynechococcus vestitus*). We demonstrate that the low-energy states discussed in this work are excitonically coupled states with significant charge-transfer (CT) character. Moreover, we demonstrate how an analysis of various optical spectra of PSI_3_–IsiA_18_ supercomplexes allows one to make inferences about EET from the IsiA_18_ ring to the PSI_3_ core and demonstrate that there most likely are three entry points for EET from an IsiA_6_ hexamer to a PSI core monomer, with two of these entry points likely being located next to each other (i.e., nine entry points from the full IsiA_18_ ring to the PSI_3_ trimer). We anticipate that the data discussed below will stimulate further research in this area, providing even more insight into the structure-based models of these important cyanobacterial photosystems.

## 2. High-Resolution Site-Selective (Low-Temperature) Frequency-Domain Spectroscopies

The HB/FLN/∆FLN and SPCS spectroscopies discussed below continue contributing to a better understanding of many complex biological systems [34,35,36,37,38,39,40,41,42,46,47,48]. These techniques can be best understood from the perspective of the theory of impurity centers in solids [73] (although, strictly speaking, in PCs, pigments are not ”impurities” or “dopants”, as they are placed there by nature, this theoretical framework still applies) and they are particularly effective at cryogenic temperatures, where one can freeze out many degrees of freedom, decrease thermal broadening effects (e.g., pure dephasing) and lower the rates of thermally induced spectral shifts. The best way to provide a complete picture of a very complex biological system is measuring various types of optical spectra, modeling them in concert and (if available) comparing time-domain and frequency-domain results. Before we present recent advances made in the understanding of the EET, electron transfer, and red antenna states of various cyanobacterial PSI and related systems, including PSI supercomplexes (PSI_3_–IsiA_18_) and isolated IsiA_1_ monomers, we briefly review several site-selective techniques.

### 2.1. Single Photosynthetic Complex Spectroscopy (SPCS)

We introduce this technique first in order to discuss some relevant concepts in a simpler manner. The spectrum of a single pigment molecule in a solid (including protein) consists of a so-called zero-phonon line (ZPL; purely electronic transition) and various vibrational sideband features building upon the ZPL. These include phonon sidebands (PSB; due to delocalized phonons of the protein environment), as well as features due to vibrational degrees of freedom of the pigment molecule itself. The width of the ZPL depends on the lifetime of the excited state, including radiative lifetime, “pure dephasing” time, and/or energy transfer or charge transfer time. ZPL is usually a Lorentzian function with a homogeneous line width *Γ_hom_* determined by

**Figure 1:**
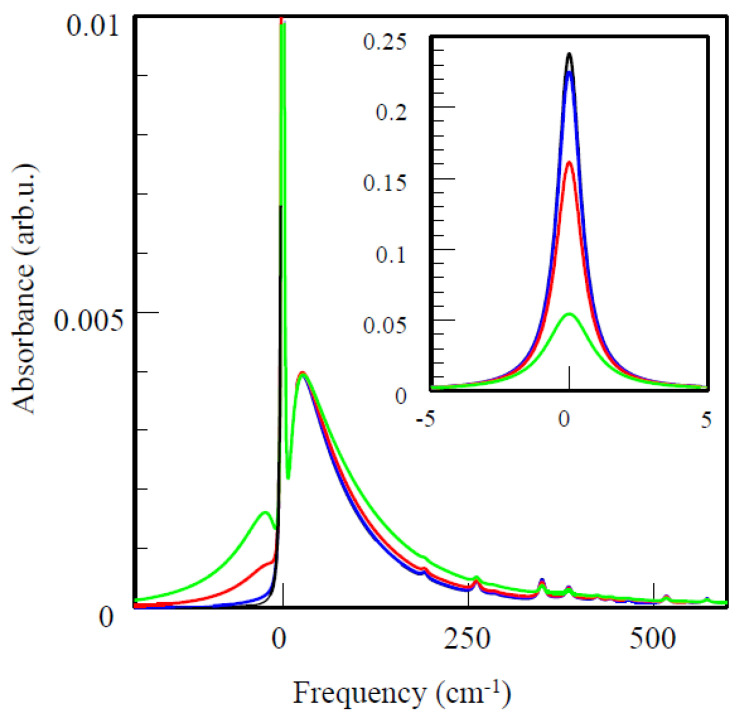
Single-molecule absorption spectrum and its temperature dependence. In the main frame, the ZPL is truncated to provide a clearer view of the PSB and Chl molecule vibrations; the insert depicts the ZPL part. The temperature increases from black to green, resulting in ZPL broadening and the increase of the PSB contribution to the spectrum. See text for more details.

*Γ_hom_* (cm^−1^) = (1/2*πcT*_1_ + 1/2*πcτ_EET_*) + 1/*πcT*_2_*(*T*) ≈ 1/2*πcτ_EET_*, (1)

where *T*_1_ is the radiative lifetime; *T*_2_* is the “pure dephasing” time, which (at *T* = 5 K) is very short in comparison to *T*_1_; *c* is the speed of light in (cm s^−1^); and *τ_EET_* is the energy transfer (EET) time. In RCs, *τ_EET_* may be replaced with *τ_ET_*, electron transfer time (also known as primary charge separation time). In pigment–protein complexes, *τ_EET_*_,_ *τ_ET_* << T_1_, *T*_2_* [34,36]. Figure 1 shows a simulated single-molecule absorption spectrum, as well as its temperature dependence. The curves contain ZPL and PSB peaked at about 25 cm^−1^ and additional small peaks due to vibrations of the Chl molecule. The black curve was obtained for *T* = 5 K, and the temperature increased from black to blue to red to green. The total area under the curve is normalized to one on the frequency scale. With the increase of temperature, the ZPL broadens due to *T*_2_*(*T*), and its relative contribution to the spectrum decreases. Both Stokes and anti-Stokes components of the PSB grow with temperature. At still higher temperatures, the ZPL and PSB would merge. Thus, frequency-domain methods feature the best resolution at cryogenic temperatures, where ZPLs are the narrowest. The l-ph coupling strength *S* can be defined via relative areas under the ZPL and the PSB as *I_ZPL_*/(*I_ZPL_* + *I_PSB_*) = *exp*(−*S*(*T*)). Isolated Chls in antenna complexes usually exhibit weak to moderate el-ph coupling [34] (*S* < 1; *S* = 0.7 in Figure 1), while the lowest states of strongly coupled Chl clusters, including various special pairs of the RCs, may exhibit strong el-ph couplings with *S* up to 5. In the latter case, ZPL might be hard to resolve even at 5 K. 

In macroscopic samples, one needs to also consider the degree of disorder of the environment. In a perfect crystal, all local environments of the pigment molecules will be identical, and this will result in identical transition energies (defined here as ZPL energies) of the pigments. In somewhat disordered solids, including proteins, transition energies will vary somewhat from one individual PC to another, giving rise to so-called inhomogeneous broadening. The latter prevents observation of the narrow ZPLs. The key advantage of SPCS [40,46,47,48] is that this approach entirely eliminates the problems of inhomogeneous broadening and sub-ensemble averaging (see SHB and FLN sections below) and allows one to investigate the properties of individual complexes and of individual molecules within one complex. The disadvantages of SPCS boil down to high light intensities required for single molecule detection, which could result in triplet formation, saturation, local heating/unknown sample temperature, and inability to detect all light-induced spectral shifts as fast as they may occur. Multiple spectral shifts occurring faster than the time scale of an SPCS experiment manifest as ZPL broadening that may result in errors in determining line widths, EET times, or shift rates. It is also not clear if some single-complex sample preparation procedures affect the shape of the complexes [74].

**Figure 2:**
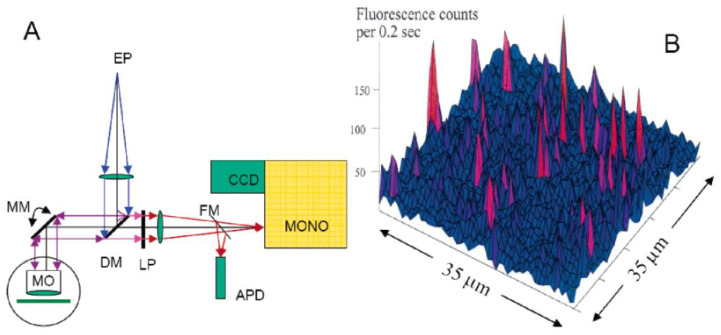
(**A**) Scheme of a confocal microscope used for SPCS. EP is excitation pinhole, DM is dichroic mirror, MM is motorized mirror, MO is microscope objective, LP is long-pass filter, and FM is flipping mirror. APD itself and the monochromator’s slit were used as detection pinholes. (**B**) Raster-scan image of the thin film containing single PSI complexes from *Synechocystis* PCC 6803 (red peaks) obtained by varying the orientation of the motorized scanning mirror. Fluorescence was collected with a 180 μm-diameter avalanche photodiode used as a pinhole. Complexes were excited with 250 nW/μm^2^ at 680 nm, and fluorescence was collected at λ > 700 nm. *T* = 10 K. Reprinted with permission from Ref. [66] Copyright 2007 American Chemical Society.

From a technical standpoint, the vast majority of SPCS experiments are based on the use of commercial or, especially at low temperatures, home-built (microscope objective in the liquid helium environment right next to the sample) confocal fluorescence microscopes [34,40,46,47,48]. A small amount of diluted sample is spin-coated onto a suitable substrate, ensuring that only one PC at a time is present in the diffraction-limited volume selected by the microscope objective. After such selection has been achieved, the fluorescence or fluorescence excitation spectra of single pigment–protein complexes are measured [34,46]. Figure 2 presents the schematic of a typical SPCS setup and the image of the spin-coated film containing PSI from [66]. 

Although significant progress has been made in the studies of single pigment–protein complexes (see [34,40,46,47,48] for reviews), we believe that much remains to be done. So far, the SPCS was employed in studies of LH2 antenna complexes of purple bacteria at low temperatures (Refs. [46,74,75,76] and many others) and at room temperature [77,78,79], LHCII peripheral antenna complex of PSII [80,81,82,83], PSII core [84], and, particularly relevant for this review, on cyanobacterial PSI [66,85,86,87,88,89,90,91,92]. In all those systems, light- and thermally induced spectral shifts have been observed, which provide information on low-temperature protein dynamics and energy landscapes and serve as the basis of the following techniques. Very recently, room-temperature single-complex data also emerged for the PSI_3_–IsiA_18_ supercomplex [93].

### 2.2. Hole-Burning (HB) Spectroscopies

As stated above, shifts of the ZPL are universally observed in pigment–protein complexes and other amorphous solids. These shifts are either light-induced or light-independent (both types can involve thermally induced barrier-hopping or quantum-mechanical tunneling). As shown in [94], light-induced shifts are by far more likely under realistic conditions of the optical spectroscopy experiments, especially the SPCS ones requiring higher excitation intensities. The simplest model capable of explaining these spectral shifts is the two-level system (TLS) model, where the energy landscape of a protein is represented by a double-well potential (as a function of some generalized coordinate). This model was first developed to explain anomalous low-temperature thermal properties of glasses [95,96] and was later adopted for hole-burning spectroscopy, including that applied to proteins. The model implies the existence of two conformational sub-states corresponding to slightly different optical transition frequencies of the pigment embedded in the protein. The extension of this model involves multi-level systems, MLS, with more than two wells.

**Figure 3:**
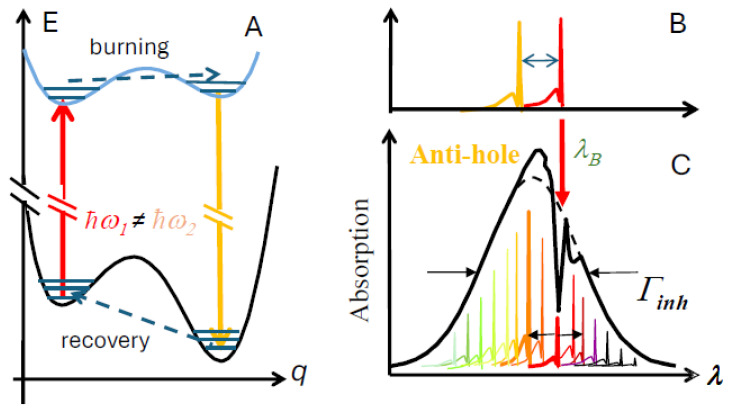
(**A**) Mechanism behind the single molecule line shifts and NPHB. Before absorbing a resonant photon, the pigment–protein system is assumed to be in the left well. Upon absorbing a photon, the system finds itself still in the left well but in the excited electronic state. The barriers are lower in the excited state, and the system has a reasonably high chance to cross the barrier into the right well within the ∼ns lifetime of the excited state. Subsequently, the pigment returns to the ground electronic state. The pigment’s transition energies are different for the left and right wells, i.e., a spectral shift is observed, as indicated in (**B**) Single-molecule spectral shifts. Note that single-molecule absorption spectra shown here contain ZPL and PSB. (**C**) When a macroscopic sample with an inhomogeneously broadened absorption spectrum based on site distribution function (SDF) of width *Γ_inh_* is illuminated with a narrow-band laser at *λ_B_*, a resonant spectral hole is formed as a result of multiple molecules’ lines shifting out of resonance with the laser. These shifted lines contribute to the NPHB “antihole”. Shifts in both directions are possible, but red-shifted molecules can be burned again via their PSB. Therefore, the antihole is usually, on average, blue-shifted. The system may return to the original (left) well, but it will take much more time than in the excited state, as the ground-state barriers are much higher.

Figure 3 presents the TLS-based mechanism behind spectral line shifts in optical experiments. Let us assume that the pigment–protein system originally resides in the left well (configuration 1). The pigment may be excited by resonant laser illumination, and then some small structural change in the protein local environment may bring the system to configuration 2 while the pigment is in the excited electronic state. At low enough temperatures, below 10 K, the barriers, except the lowest ones, are crossed predominantly by tunneling [97]. Eventually, the pigment–protein system returns to the ground electronic state and is trapped in configuration 2. The parameters of the ground state energy landscape determine how long it will take for the system to return to configuration 1. Note that the barriers are much lower when the pigment is in the excited electronic state, so they could be crossed with reasonable probability within the nanosecond or sub-nanosecond excited state lifetimes, but recovery (crossing barriers in the electronic ground state) may take hours or even days at 5 K. The barriers must be lower in the excited state, as otherwise, the NPHB holes would recover as fast as they burn and persistent NPHB would be impossible.

To perform HB experiments, the sample is cooled down to liquid helium temperatures. A tuneable continuous wave (CW) narrow-band dye-, Ti-sapphire, or diode laser is required. Alternatively, a fixed-wavelength laser could be combined with a sufficiently high-resolution VIS–NIR spectrometer. Visible-range-capable FT spectrometers could be used, except in scenarios where white-light illumination causes charge separation (which is the case for PSI) and keeping RC in a reduced state is required for some reason (see Section 4.3). A CW laser beam with a very narrow bandwidth (narrower than the relevant ZPL) probes a sub-ensemble of the pigment molecules, which have a ZPL resonant with the laser frequency. The HB spectra are the difference in absorption (or fluorescence excitation) spectra before and after illumination by the laser. If the absorbance changes are preserved after illumination, the HB spectrum is said to be persistent; otherwise, the HB spectrum is called transient (see below). Persistent HB can be further divided into photochemical and non-photochemical (NPHB) varieties. In the case of NPHB, the pigment molecule does not undergo any chemical reaction, but its immediate environment experiences some rearrangement (see Figure 3; for more details see [34,36]). HB spectroscopy can provide lifetimes of the zero-point level of S_1_(Q_y_)-states due to EET and/or ET. The lifetimes can be determined via the widths of shallow zero-phonon holes (ZPH) burned at low fluence, with *Γ_hom_* = 1/2 ZPH width [34,36,37]. In addition to the ZPH and the antihole, the NPHB spectrum has two side-holes. The one in the blue with respect to the ZPH is due to the PSB of the ZPH (see Figure 1) and is called “real PSB”. The one in the red is due to ZPLs burned via their PSB and is called “pseudo-PSB” [34].

**Figure 4:**
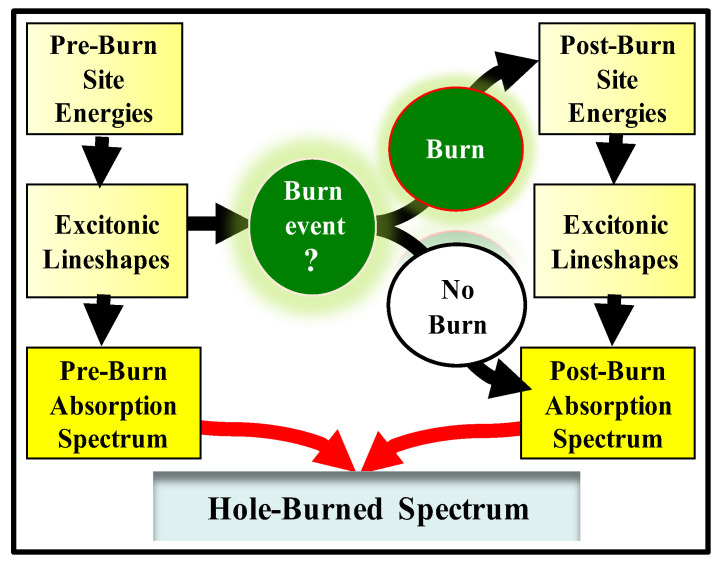
Modeling of HB spectra in excitonically coupled systems. Reprinted with permission from Ref. [99] Copyright 2011 American Chemical Society.

There are two main ways to model spectral hole-burning in pigment–protein systems. In the first approach, disorder is accounted for by choosing pigment site energies randomly from usually Gaussian distributions, which may differ for pigments at different locations within the complex, Monte-Carlo style. Each randomly generated set of pre-burn site energies corresponds to a particular realization of disorder in an ensemble representing a macroscopic sample. The site-energies, along with the inter-pigment interaction energies, determine the shapes of various optical spectra of the PC, as well as the details of the EET processes [54,61,98]. For each complex, the Hamiltonian generated with the pre-burn site energies and coupling constants is diagonalized and the excitonic line shapes from all the states are then summed to produce the pre-burn absorption spectrum (or, more precisely, the site distribution function, SDF). Band shapes are calculated using either the (static) single-site spectrum (convolution method [54,55]) or a more advanced approach including delocalization and energy transfer effects (after the pre-burn spectrum is generated, a decision is made whether to “burn” the selected molecule: the probability of that event is determined depending on whether given molecule is excited by the laser, how much it contributes to a given excitonic state, and whether a particular excitation event leads to HB (NPHB yield is much smaller than one, but the transient HB yield may approach one, if transient holes are due to charge separation; see below)). If the molecule is declared “burnt”, spectral shift is picked from an appropriate distribution. Finally, the post-burn absorption spectrum is calculated using the new post-burn site energies as input parameters; the difference between the post-burn and pre-burn absorption spectra is the HB spectrum. Both resonant and non-resonant (transient and/or persistent) holes can be calculated [6,34,54,99]. The transition energies of the pigments inside the pigment–protein complexes are determined by pigment–protein interactions. Note that the EET processes are affected not only by the static structure of the complex, but also by the protein dynamics. The light-induced shifts of the pigment site energies in NPHB spectroscopy, which are determined by the properties of the protein energy landscapes, affect the positions and oscillator strengths of excitonic states [54,55,56,57,99]. The light-induced shifts influence the shapes of resonant- and non-resonant HB spectra [34,54,55,56,57,65,67,71] and CD spectra. The calculated band shapes for both pre-burn and post-burn spectra are affected by the single-site spectrum and depend on the temperature (T), el-ph coupling strength *S,* and “one-phonon profile” (equivalent to phonon spectral density *J*(*ω*)) [44,100] up to a temperature-dependent weight on the one-phonon profile) [34,44,100]. Note that the one-phonon profile at low temperatures can be accessed by HB experiments, though the most reliable shape is obtained by ∆FLN experiments, a combination of HB and FLN experiments; see below [34,42,43]. A schematic for the numerical simulations of HB spectra in excitonic systems is shown in Figure 4.

Another approach to modeling NPHB relies on the so-called NPHB master equation and is mostly used when the focus of the study is on spectral diffusion and respective energy landscapes in proteins at low temperatures (see Figure 3). (Bacterio)chlorophyll-protein complexes in particular are ideal model systems to study protein energy landscapes with optical spectroscopy, as pigments are built into protein by nature (and in a large variety of local environments) without any additional chemical manipulations or genetic engineering that might alter the original protein. Several classes of HB experiments probe different aspects of the protein energy landscapes. Experiments on hole evolution during the burning process, in particular, hole growth kinetics measurements, probe the distribution of barriers in the excited electronic state of a pigment-host system [97,101,102,103,104], denoted by the blue curve in Figure 3A. Hole recovery and thermocycling experiments provide information on ground-state energy landscape properties and spectral diffusion mechanisms (see [97,104] and references therein). The NPHB master equation describes the absorption spectrum after illuminating the sample at *ω_B_* with photon flux *P* for time *t*:

(2) $$\begin{array}{l} D(\Omega ,t)=1.5\int d\omega L(\Omega -\omega )G(\omega )\int d\lambda f(\lambda )\\ \;\;\;\;\times \int d\alpha \sin\alpha \cos^{2}\alpha e^{-P\sigma \varphi (\lambda ,\tau_{fl})L(\omega_{B}-\omega )t\cos^{2}\alpha } \end{array}$$

Here, *σ* and *ϕ* are the integral absorption cross-section and the NPHB quantum yield, respectively.

(3) $$\varphi (\lambda ,\tau_{fl})=\frac{\Omega_{0}\exp(-2\lambda )}{\Omega_{0}\exp(-2\lambda )+\tau_{fl}^{-1}}$$

In the simplest approximation, $\lambda =d\sqrt{2mV}/\hbar$, where *d* is the thickness of the barrier, *V* is its height, and *m* is the mass of the tunneling entity. *f*(*λ*) is the distribution of *λ* and *τ_fl_* is the fluorescence lifetime. *L*(*ω_B_ − ω*) is the single site absorption profile (single-molecule spectrum). Ω_0_ is the attempt frequency, and *α* is the angle between the laser polarization and the transition dipole moment of the pigment. *G*(*ω*) is the SDF, which is Gaussian before burning, and it describes the probability of finding a pigment with a ZPL at a given frequency. This is the same function that is used to generate the site energies in the Monte-Carlo approach above. For details and application to various photosynthetic systems, see [34,94,104] and the references therein. Recently, a combined Monte-Carlo/NPHB master equation approach has been introduced, but due to the high computational cost, it has so far been limited to a small number (2–3) of interacting molecules per complex [105].

The discussion above pertains to an isolated pigment inside a protein (only resonant HB is possible). However, intramolecular relaxation and EET will occur for excitation of higher lying electronic states and molecular aggregates, respectively. The mechanism for resonant HB is then in competition with the excited state relaxation pathways and the quantum yield for HB resonant with the laser decreases. In the cases where EET or ET are present, *τ_EET_*^−1^ or *τ_ET_*^−1^ should be added to the denominator of Equation (3), and for a sufficiently fast depopulation due to EET, a resonant NPHB may not be observable for higher-energy excitonic states. In this case, a non-resonant HB spectrum (see Section 4 and Section 5) can be measured with a broad hole in the spectral range of the lowest energy (emitting) state, which undergoes some HB before relaxation to the electronic ground state. Such non-resonant spectra show the spectral position and shape of the lowest energy trap state [54,65,67,71].

The generation of transient HB spectra requires the presence of a third, relatively long-lived state [34,37]. That is, the pigment transitions from the excited singlet state into a triplet state [106,107] or is converted photochemically to another long-lived (μs to ms range) product (e.g., a charge-separated state in the case of the RCs [37]), leaving a transient hole in the absorption spectrum with a ZPH at the excitation frequency (for resonant HB) and with the shape defined by the el-ph coupling parameters. In this case, the pigment’s ground state is depopulated for the lifetime of the long-lived state, and the spectral hole will be observable only for the duration of this lifetime. One advantage of transient holes is that they do not possess an antihole interfering with the real phonon sideband feature of the original hole. The transient holes are defined as the difference between the absorption spectra measured while the excitation is on and off, usually after the persistent hole component has been saturated [34,37].

### 2.3. Fluorescence Line Narrowing (FLN) Spectroscopy and Delta FLN (∆FLN)

**Figure 5:**
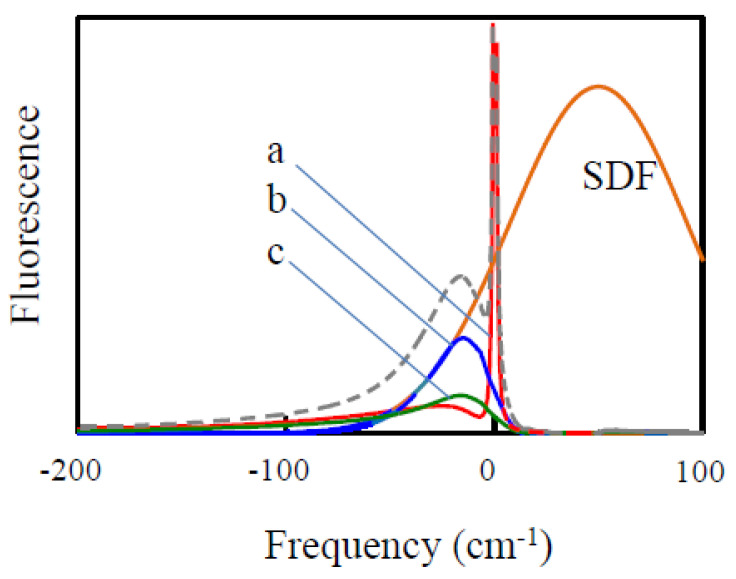
The components of the FLN spectra. Red, a: emission spectrum of the molecules excited via ZPL, containing ZPL and real PSB. ZPL is truncated. Mirror image of the 5 K absorption spectrum from Figure 1. Blue, b: “pseudo-PSB”, the ZPLs of the molecules excited via their PSB. Green, c: real PSB of the pseudo-PSB. Dashed gray curve is the sum of the above contributions. Orange curve is the SDF of the emitting state.

The fluorescence line narrowing technique was originally developed to obtain the el-ph coupling parameters (e.g., PSB shapes), especially in systems where HB was not possible for some reason. Like HB, FLN involves a narrow-band laser. The fluorescence spectrum is measured while the sample is illuminated at some fixed wavelength within the emitting state/band. ∆FLN is the further development of the FLN technique, where FLN is combined with spectral HB, and FLN spectra are measured before and after hole burning (more details can be found in [34,42,43]). It has been demonstrated [34,42] that the PSB part of the ∆FLN spectrum is, in a very good approximation, the PSB shape (including multi-phonon processes). The accuracy of the determination of the Huang–Rhys factor *S* strongly depends on one’s ability to suppress scattered excitation light in the ZPL region without suppressing the ZPL contribution to fluorescence. ∆FLN with excitation into the higher excitonic states can also be used, for instance, to determine inter-pigment couplings and EET rates, especially in a weak coupling regime [42]. Figure 5 explains the main contributions to the FLN spectrum. Red curve (a) represents the emission of the molecules excited via their ZPLs. It is proportional to a single-molecule emission spectrum containing ZPL, PSB, and intra-molecular vibration components (outside of the range of the figure). Blue curve (b) represents the ZPL contribution to the emission of the molecules that were excited via their PSB. It is limited to the low-frequency side by the edge of the SDF (the probability of finding molecules with ZPL at various energies; orange). This is essentially the analog of the pseudo-PSB component in the NPHB spectra. The green curve is the PSB contribution to the emission of the same molecules. The dashed curve is the sum of all the above contributions to emission. In the case of ∆FLN, additional spectral selection takes place, as components (b) and (c) are now based not on the full SDF, but on the sub-SDF of the molecules that have been hole-burnt. The latter sub-SDF is dominated by the ZPH contribution (resembling (a)). As a result, components (b) and (c) are suppressed in the ∆FLN spectra, the latter remaining dominated by the contribution (a). Thus, ∆FLN yields, in a good approximation, single molecule spectra including all vibrational contributions.

In summary, information provided by the HB and ∆FLN spectroscopy includes the following [34,37]: (1) lifetimes of the zero-point level of excited S_1_(Q_y_)-states due to EET and/or ET, as determined by the widths of ZPH; (2) *Γ_inh_* values, typically ~50–200 cm^−1^, derived from the ZPH action spectrum, i.e., the envelope of multiple ZPHs burned at different wavelengths under constant irradiation dose conditions; (3) el-ph (protein) coupling parameters (including both shape and *S*, defined in the low-temperature limit as the integrated area of the spectral density) for phonons and/or pseudolocalized phonon frequencies; and (4) various parameters of the relevant protein energy landscapes. As far as comparisons between frequency-domain and time-domain spectroscopies go, in principle, they provide the same information about EET and ET rates, but their experimental considerations could be quite different. For instance, in time domain measurements, phase stability is of great importance, whereas in frequency domain measurements, achieving high resolution and obtaining the single-site line shape are the main considerations. One should be aware that NPHB tends to preferentially select molecules with the longest lifetimes (due to *τ_EET_* affecting the denominator of Equation (3)), while time-domain experiments may miss the longest-time decay components. Thus, EET times obtained using NPHB historically tended to be somewhat longer than their time-domain counterparts. These discrepancies may be addressed by proper NPHB modeling incorporating the distributions of ZPL widths due to the distributions of EET times [94].

## 3. Structure and Function of PSI: Plants vs. Cyanobacteria, *Synechocystis* PCC 6803 vs. *Thermosynechococcus elongatus*; Supercomplexes in Iron-Deficient Environments

**Figure 6:**
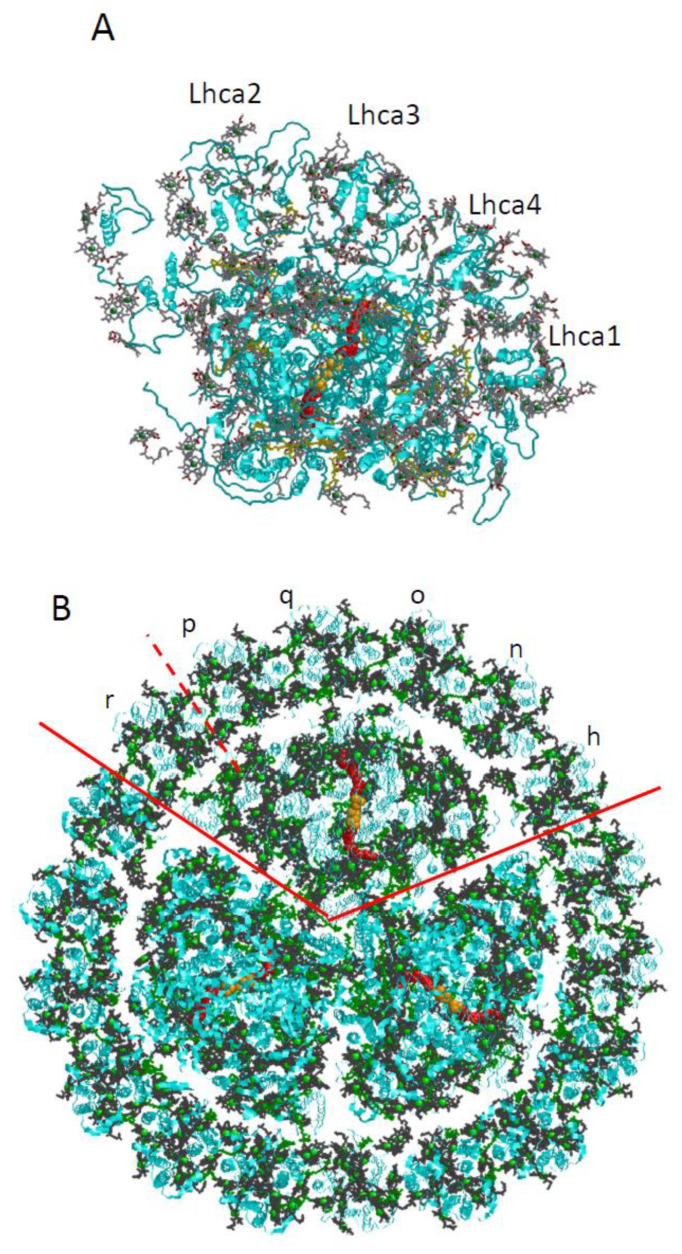
(**A**). Structure of plant PSI (monomeric, with peripheral antenna complexes; based on 3LW5.pdb structure from the Protein Data Bank). Chls are in gray, with central Mg atoms in green. Protein is in cyan. Orange iron–sulfur clusters and red quinones indicate the location of the RC. (**B**). Structure of cyanobacterial PSI (trimeric, with an additional ring of IsiA antenna proteins that are present only in the case of iron-deficient growth conditions; based on cryo-EM structure 6NWA.pdb). IsiA subunits labeled r–h form the “hexamer” referred to in Section 5. Figures were created with RasWin 2.7.4.2.

Photosystem I is one of the two major protein complexes involved in oxygenic photosynthesis. Its main function is to catalyze light-driven electron transport across the thylakoid membrane. Multiple detailed 3D structures of PSI are available both for cyanobacteria and plants [108,109,110,111,112,113,114,115,116,117,118,119,120,121,122,123,124,125]. PSI is present in monomeric form in plants [111,112,113,114,115,116], while in cyanobacteria, it is usually trimeric [108,109,110,115,116,117,118,119]. In cyanobacterial PSI_3_, peripheral antennas are usually not present, except in the case of iron stress [120,121,122,123,124,125,126]. (Attachment of phycobilisomes to PSI has been reported in *Anabaena* sp. PCC 7120 [127].) The antenna Chls are mostly located in the main core subunits PsaA and PsaB that are surrounded by several small transmembrane proteins that stabilize the complex and facilitate trimerization in the case of cyanobacterial PSI_3_ [128]. In all organisms, the RC of PSI (see Figure 6) is an integral part of the PSI core. The primary electron donor in PSI (P700) is most likely the “special pair”, a Chl *a*/Chl *a*’ heterodimer located close to the lumenal side of the membrane (eC1_A_/eC1_B_), but there is also some spectroscopic evidence that charge separation can initiate at the eC2/eC3 pair in the electron transport chain of PSI [129]. The highly efficient EET within the antenna network and to the RC is followed by charge separation between P700 and primary acceptor, A_0_ (Chl *a*). This transfer involves another Chl *a*, the “accessory Chl”. More information on the role of accessory Chl can be found in [4,129,130,131,132,133]. The electron is further transferred to the intermediate electron acceptor A_1_ (phylloquinone; red in Figure 6) [119], then to the Fe_4_S_4_ cluster F_X_ and to the terminal electron acceptors F_A_ and F_B_ (also Fe_4_S_4_ clusters, orange in Figure 6). The P700 absorption peaks at around 700 nm [134,135,136,137].

In both plants [49,138,139,140,141] and cyanobacteria [65,66,67,68,69,70,71,85,86,87,88,89,90,91,92,93,142,143,144,145,146,147,148], some antenna chlorophylls absorb at wavelengths significantly longer than 700 nm. These Chls are known as “red chlorophylls” and respective bands (also known as “red antenna states”), and their number, peak absorption, and emission wavelengths vary between species. The relatively long wavelength of these states is attributed to the combination of the red shifts of the site energies (transition energies in the absence of inter-pigment coupling) of the contributing Chls due to particular interactions with the protein environment, as well as to strong electrostatic interactions within the groups of closely spaced pigments, leading to excitonic splitting. There are also indications that some red states may possess a charge transfer character [65,69,70,71]. At physiological temperatures, these states can transfer energy uphill to the P700 and they serve to extend the wavelength range of harvested light. At cryogenic temperatures, they act as competing energy traps [67,142,145]. Note that in plants, the red states belong to the peripheral Lhca1–4 light-harvesting complexes [138,139,140,141] that are not present in cyanobacteria. The lowest-energy states of PSI from several cyanobacteria were identified, and competing assignments of these states to particular groups of the strongly coupled Chls were proposed [63,65,66,67,71,86,87,91,98,142,144,146,147,148,149,150,151,152].

**Figure 7:**
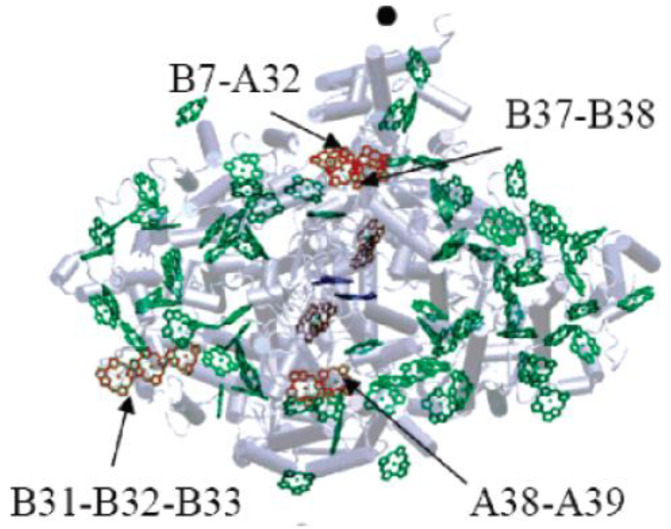
Structure of the PSI from *T. elongatus*. Only one monomer is shown; figure from [148] was used as a starting point for further modifications. Chl clusters that are most widely considered the origin of the red antenna states in PSI of *T. elongatus*, originating from [146] are highlighted in orange. Black dot indicates the approximate location of the C_3_ symmetry axis of the PSI trimer. Reprinted with permission from Ref. [67] Copyright 2016 American Chemical Society.

The groups of Chls most frequently considered as the origin of the “red states” in cyanobacteria are shown in Figure 7, which is based on *T. elongatus’* structure [108,109]. B37–B38 and A38–A39 are located on the stromal side of the complex, while B31–B32–B33 and B7–A32 (+A31 + B6) clusters are located on the uminal side. B7–A32 and B37–B38 are located close to the C_3_ symmetry axis of the PSI trimer. The peripheral location of the B31-B32–B33 Chl trimer suggests that it likely makes an independent trap, separate from the other red states (Figure 7), which are significantly closer to P700. Until recently, it was believed that *Synechocystis* PCC 6803 possesses two pools of red Chls, C706 and C714 [65,70], while *T. elongatus* has at least three red antenna states, C710 (sometimes also labeled C708; with a distinct relatively narrow band), C719, and C715 [71,85,86,87,88,89,90,91]. States are labeled according to their peak absorption wavelengths. The lowest-energy states of *T. elongatus* and *Synechocyctis* PCC 6803 exhibit some similar properties (very strong el-ph coupling, large pressure-induced spectral shifts, and strong Stark effect [65,66,69,71]), which suggests that these states may originate from a Chl cluster whose structure is preserved across species and that these states possess significant CT character. (Similar conclusions attributing very strong el-ph coupling and large pressure-induced shifts to CT states can be made for the red-most states of plant PSI, which belong to the peripheral antenna (see Figure 6A) [49,138,139]).

However, C719 of *T. elongatus* and C714 of *Synechocystis* PCC 6803 also exhibit some significant differences (e.g., in how their fluorescence is affected by the oxidation state of P700 [67,142]), and complete certainty concerning the origins of each of these red states has not yet been reached. Theories with different levels of complexity (dipole–dipole approximation is not applicable in the case of inter-pigment distances comparable to the size of the Chl macrocycle, i.e., smaller than about one nanometer [98,146,147,148,149,150,151]) assign the strongest inter-pigment couplings to different groups of pigments. There definitely are more Chl *a* clusters with strong inter-pigment couplings (|*V_ab_*| >100 cm^−1^) than there are “red antenna states” [67,98,134,146,147,148,149,150,151]. Moreover, there is no agreement concerning the site energies (transition energies in the absence of inter-pigment interactions) of the Chls in PSI [98,146,147]. Therefore, assignment of the red states still requires additional comparisons between structural and spectroscopic data. Until recently, most of these comparisons were performed for *T. elongatus*, for which the X-ray structure has been available since 2001 [108,109,110]. This structure was eventually further refined using density functional theory (DFT) [153]. Recently, comparable-quality structural data for *Synechocystis* PCC 6803 became available, confirming that the analog of B33 Chl of *T. elongatus* is missing. [118,119]. The current assignments of the red states of *T. elongatus* may be summarized as follows: it was known for a while, even before the structure of PSI became available in 2001, that red states are affected by trimerization/monomerization of the PSI core. In the case of *T. elongatus*, the most affected states are C715 and C719 [131], indicating that these states are likely due to B37–B38 and B7–A32. The fluorescence of PSI from *T. elongatus* is strongly affected by the oxidation state of P700, indicating that the C719 cluster is close to the special pair [67,142], making B7–A32 the most likely candidate, with B37–B38 and A38–A39 less likely but still possible. DFT and INDO calculations suggest that neither B37–B38 nor A38–A39 are particularly ”red” [147] and there is also experimental evidence that A38–A39 absorbs elsewhere [136]. Comparison of satellite hole structures with the simulated spectra of the red Chl clusters suggests that C719 originates from a cluster with the lowest excitonic state being the strongest [67], again resulting in preference for B7–A32 over B37–B38. Most studies agree that B31–B32–B33 is one of the red Chl clusters, with some attributing it to C719, which, however, would be in strong disagreement with [137,142]. It was reported in [66] that C715–C719′s EET time is around 5 ps, indicating that C715 is likely due to B37–B38 (close to B7–A32) and not due to B31–B32–B33 (far from B7–A32). This leaves us with B31–B32–B33 as the origin of the C710 state. These assignments are included in the first table in Section 4. (The fourth red pool for *T. elongatus* with the lowest state peaked at about 712 nm has been tentatively proposed in [67] based on the differences in non-resonant hole structures obtained with burning at different wavelengths (see Section 4). It was assigned to B14–B15, one of the Chl clusters also considered in [134].)

Recently, a high-resolution structure has been determined for the PSI of *Gloeobacter violaceus* that does not possess absorption bands beyond 700 nm and emits at about 695 nm at low temperatures [152]. This PSI lacks seven Chls compared to *T. elongatus* (six compared to *Synechocystis* PCC 6803), with two of them associated with PsaA and PsaB subunits; thus, two of the red states present in other cyanobacteria have been assigned to B31–B32 (or B31–B32–B33) and A12–A14 [152]. The latter cluster could indeed be the origin of a red state according to inter-pigment couplings and site energies from [98,147] and is located on a periphery of the trimeric core. On the other hand, the number of the red states observed in *Synechocystis* PCC 6803 and *T. elongatus* is larger than the number of Chls missing from the PsaA and PsaB subunits of *Gloeobacter violaceus*, thus the absence of these Chls cannot be the only factor affecting the red states. Conversely, the presence of any of these Chls does not guarantee that the respective cluster gives rise to a red state.

More light on the number of red Chl pools and their identification was shed by the studies of PSI mutants from *Synechocystis* PCC 6803 [63]. The wild type (WT) *Synechocystis* PCC 6803 does not possess the B33 pigment, which makes comparisons pertaining to the B31–B32–B33 Chl cluster particularly interesting. The B31–32 dimer or B31–B32–B33 trimer may contribute to light harvesting in the far-red region of the spectrum. It is also possible that this Chl cluster participates in photoprotection. While the B31–B32 dimer or B31–B32–B33 Chl trimer do not focus energy on P700, they may play a role in EET to PSI from the IsiA_18_ antenna in a PSI_3_–IsiA_18_ supercomplex [64]; see Section 5 for details. The recent structure of the PSI_3_–IsiA_18_ supercomplex [124] showed that the predicted lowest-energy Chls of the IsiA ring are likely located close to the B31–B32 dimer. Each PSI monomer also coordinates 22 carotene (Car) molecules spread across the antenna; carotenoids arguably play a role in photoprotection [154]. They closely interact with approximately two-thirds of the antenna Chls and a Car is located in the vicinity of each of the most likely red sites in PSI outlined above (Figure 7). Thus, close interaction between red Chls and Cars may be an important mechanism for photoprotection in PSI.

**Figure 8:**
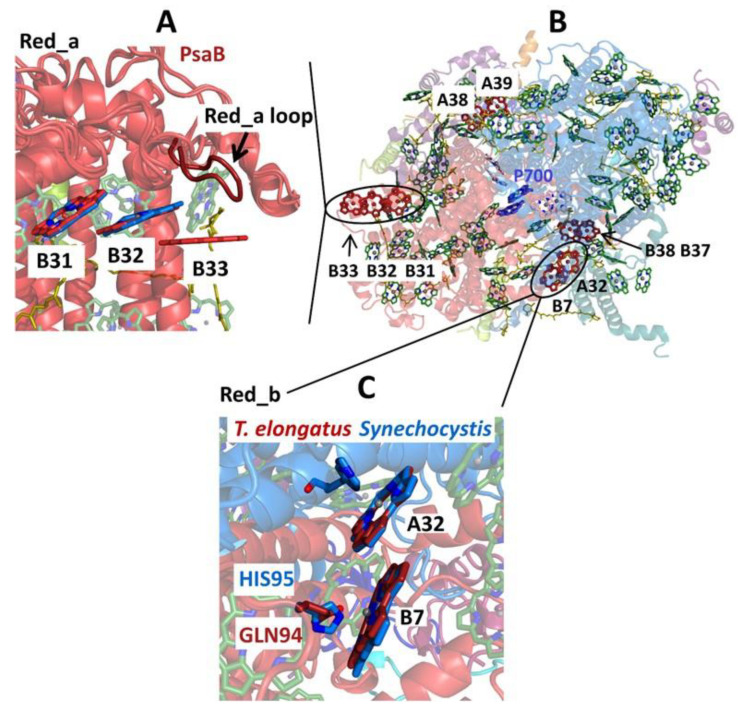
Arrangement of Chls in PSI and illustration of mutations used in this study. (**A**). The Red_a mutation involves an insertion of four amino acids (marked as Red_a loop) coordinating the additional Chl B33. (**B**). Overall arrangement of PSI from *T. elongatus*; likely red-absorbing Chls are highlighted in red [109,146]. P700 in blue is shown in the center. (**C**). Overlaid structures from *T. elongatus* and *Synechocystis* PCC 6803 from [119] in the vicinity of the B7–A32 cluster; in Red_b mutant of *Synechocystis* PCC 6803 (blue), His95 was mutated to Gln95 (Gln94 in *T. elongatus*, red). Reprinted with permission from Ref. [63] Copyright 2020 American Chemical Society.

As stated above, to provide more insight into the red-Chl traps, several mutant PSI were studied recently [63]; some structural details of these PSI mutants are depicted in Figure 8. It shows the arrangement of strongly coupled Chl clusters likely responsible for the low-energy states (traps). Trimer B31–B32–B33 in the Red_a mutant is shown as red; A38–A39 dimer, B7–A32 (+B6 + A31) cluster, and B37–B38 dimer are also highlighted for completeness. Although the Chl clusters above represent the best candidates for the red Chls in PSI, the assignment and energetics of corresponding states is not straightforward, as discussed below, in light of very recent findings. In Section 4, we discuss the frequency-domain optical spectra obtained recently for the Red_a, Red_b, and Red_ab mutants [63] that suggest that there are three red states in *Synechocystis* PCC6803. As an example, Figure 8B shows the arrangement of the RC Chls in PSI of *T. elongatus*, along with strongly coupled candidates for the low-energy states (traps) described above.

Under standard growth conditions (high concentration of iron, BG-11 medium), cyanobacterial PSI is a trimer (PSI_3_), usually without peripheral antennae. When cells grow under iron-deficient conditions instead, the cyanobacterial phycobiliprotein, Photosystem I, and Photosystem II (PSII) contents are reduced [155]. The studies on cyanobacteria grown in low-iron conditions revealed a remarkable adaptation manifested as the induction of a Chl-containing additional membrane antenna, the iron-starvation-induced protein A (IsiA) [156,157]. Once IsiA production is induced in cyanobacteria, it forms a large supercomplex consisting of the 18-mer IsiA ring (IsiA_18_) surrounding the PSI_3_ trimer [121,122,125]. The IsiA protein is similar to PsbC, the CP43 antenna protein of the core of PSII, the water-splitting and oxygen-evolving enzyme of photosynthesis. Therefore, the IsiA monomer is often called CP43′ [7,62,68,158]. However, it was recently determined that each IsiA subunit in cyanobacteria binds four more pigments in addition to the 13 Chls observed in the CP43 complex of PSII [54,124]. These four additional Chls contribute to the interfaces between neighboring IsiA subunits and the PSI_3_–IsiA_18_ interface [124], likely facilitating EET. The cryogenic electron microscopy (cryo-EM) structure of PSI_3_–IsiA_18_ supercomplex of the *Synechocystis* 6803 was solved at a resolution of 3.3 Å. Recently, Cao et al. solved the single particle structures of both PSI_3_–IsiA_18_–Fld (where Fld is flavodoxin and replaces the iron-containing ferredoxin (Fd) as the electron receptor of PSI) and PSI_3_–IsiA_18_ supercomplexes from a mesophilic cyanobacterium *Synechococcus* sp. PCC 7942 at resolutions of 3.3 Å and 2.9 Å, respectively [159]. The structure of PSI_3_–IsiA_18_ from thermophilic cyanobacterium *Thermosynechococcus vulcanus* is also known [122]. The overall structure of the PSI_3_–IsiA_18_ supercomplex (from Ref. [124]) is shown in Figure 6B. The configuration of the different IsiA monomers around PSI_3_ is somewhat flexible, and in the first approximation, the IsiA ring has C_3_ rather than C_18_ symmetry [124]. The ring around PSI_3_ acts mostly as a peripheral antenna; however, it was also suggested that the IsiA_18_ ring may act as an excess energy dissipater that helps protect cyanobacterial cells from oxidative damage [160,161,162]. The IsiA_18_ antenna in the PSI_3_–IsiA_18_ supercomplex is tightly coupled to the core antenna of PSI_3_ [158]. An earlier analysis of the low-temperature absorption spectra of PSI_3_, PSI_3_–IsiA_18_, and IsiA complexes was based on the assumption that there are just 13 Chls per IsiA monomer [68], i.e., a number that was observed in the CP43 complex of PSII [163]. However, as mentioned above, the recently solved cryo-EM structures of the complex revealed a 17 Chls per IsiA monomer [124]. This indicates that available isolation/purification procedures may result in the loss of some Chls. Recently, it has also been reported that individual PSI_3_–IsiA_18_ supercomplexes feature a fairly broad distribution of orientations of the IsaA subunits with respect to the core trimer, but high efficiency of EET from the ring to the core is nevertheless preserved. Finally, we mention that supercomplexes containing one PSI monomer and 6 IsiA units have been reported for *Anabaena* sp. PCC 7120 [123]. The same bacterium also exhibited tetrameric core complexes under some conditions [127].

## 4. Applications of Frequency Domain Methodologies to PSI and Its Mutants

Multiple papers have been published over the years that debate the composition of the low-energy states in the cyanobacterial PSI of *Synechocystis* PCC 6803 and *T. elongatus* [40,65,66,67,68,69,70,71,85,86,87,88,89,90,91,98,136,142,144,145,146,147,148,149,150,151]. More than 30 strongly coupled Chls (out of 95–96, i.e., per core monomer) were proposed at different times as possible contributors to the red states [98,146,147,148,149,150,151]. Thus, multiple assignments of the red pigments in PSI are available [62,63,66,67,71,86,87,144,146,147,148,149,150,151], and without the additional information from the studies of mutant PSI, which became available only recently, some assignments remained a matter of debate. Settling this debate is important since red pigments play a critical role in EET dynamics in all photosynthetic complexes. As stated above, in our earlier works, we suggested that the PSI complexes of *Synechocystis* PCC 6803 have two red antenna states, known as C706 and C714 [65,66,69,70], labeled according to the absorption band (or respective non-resonant HB bleach) maxima. The C714 state exhibited a large pressure-induced spectral shift, a large dipole moment change, and strong el-ph coupling (*S*), indicating a significant charge-transfer (CT) character of this trap [70,71,164], in agreement with the red-shifted fluorescence maxima observed near 721–724 nm [65]. However, the origins of the C706 state in WT *Synechocystis* PCC 6803 were initially unclear. Below, we summarize the data obtained recently via high-resolution low-temperature spectroscopies for WT PSI and its three mutants (Red_a, Red_b, and Red_ab) [63]. In the Red_a mutant, B33 chlorophyll (Chl) was added (via the insertion of four amino acids coordinating this Chl) to the B31–B32 dimer, and in the Red_b mutant, His94 (which coordinates Mg in A32 Chl within the B7–A31–A32-His94 cluster) was replaced with glutamine (Gln), making the local environment more similar to that in *T. elangatus*. In the Red_ab mutant, both mutations were introduced. Comparisons between the low-temperature absorption, emission, and resonant and non-resonant hole-burned (NPHB) spectra of these mutants shed more light on the energy/composition of the lowest energy states [63]. We confirmed that the lowest energy state in WT *Synechocystis* PCC 6803 is indeed the C714 trap, likely localized on the B37–B38 dimer, while the C719 trap in *T. elongatus* (i.e., the lowest energy state in *T. elongatus*) is contributed to by the Gln94-B7–A31–A32 cluster [63]. It was further demonstrated that addition of Chl B33 (in the Red_a mutant) and His95 → Gln95 mutation in the Red_b mutant of *Synechocystis* PCC 6803 (Figure 8) shifts the respective C706 and C707 states to longer wavelengths, affecting both the energy transfer pathways and resulting emission spectra. (Note that in the model of *Synechocystis* PCC 6803 advanced in [63], there are two red pools in WT PSI absorbing at around 706–707 nm; more details will be provided below. See also Table 1. Here, we only note that the Red_a mutation affects the C706 state, while the Red_b mutation modifies the C707 state).

### 4.1. Low-Temperature Absorption, Emission, and HB Spectra

**Figure 9:**
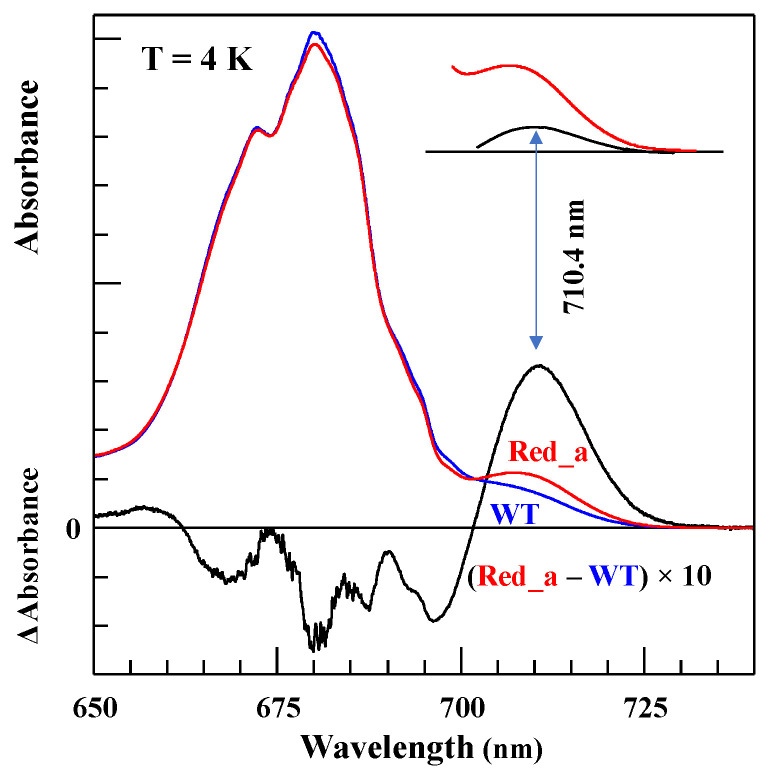
Blue and red curves are normalized (for pigment content) absorption spectra of WT *Synechocystis* PCC 6803 and its Red_a mutant, respectively. (T = 4 K, spectral resolution 4 cm^−1^). The black curve (multiplied by a factor of 10) shows the difference between the two absorption curves (Red_a minus WT PSI trimer); see text for details. Reprinted with permission from Ref. [63] Copyright 2020 American Chemical Society.

The absorption spectra of the WT *Synechocystis* PCC 6803 and respective Red_a mutant are shown in Figure 9. This mutant contains the B31–B32–B33 Chl trimer (i.e., the B33 Chl was added to the B31–B32 dimer) to mimic the trimer observed in *T. elongatus* (see Figure 8A and [63] for details). Thus, there is one additional Chl per monomer compared to WT. Both the absorption spectra in Figure 9 have a Q_y_-integrated area normalized to the expected Chl content. It can be seen that the absorption difference between the Red_a mutant and WT PSI within the 700–725 nm spectral region accounts for an additional absorption of about two Chls per PSI monomer, not one Chl as expected (see the positive band with a maximum near 710.4 nm). Based on the cryo-EM structure of the Red_a mutant PSI [165], we can exclude the possibility that more than one additional Chl was incorporated per PSI monomer; therefore, it appears that one of the low-energy states (most likely the C706 state associated with the B31–B32 dimer in WT) shifts red to ~710.4 nm upon the addition of Chl B33 and gains oscillator strength. That is, mutation converts a dimer with a relatively weak lower-energy state into a trimer with a stronger lower state, in agreement with preliminary theoretical calculations (see [67] for *T. elongatus*). The larger oscillator strength could result from stealing intensity from higher-energy states, leading (at least in part) to negative absorbance changes observed in the (black) difference spectrum between 650 and 700 nm. Note that the difference spectrum might be also contributed to by mutation-induced minor protein conformational changes and/or minor changes in the higher-energy exciton states [63,65].

**Figure 10:**
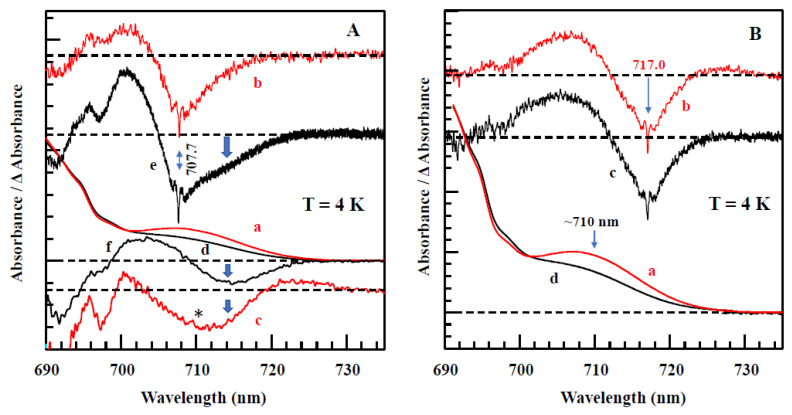
Black and red curves (in frames **A** and **B**) illustrate spectra obtained for WT PSI of *Synechocystis* PCC 6803 strain and its Red_a mutant, respectively. The corresponding absorption spectra are labeled in both frames as curves d and a, respectively. The absorption spectra are normalized in the Q_y_ region according to Chl content. Curves b and c in frame **A** (for the Red_a mutant) were obtained with *λ_B_* of 707.7 and 670.0 nm, respectively. The same types of spectra for the WT PSI (see curves e and f) are shown for comparison. (**B**). Curves b and c correspond to the HB spectra obtained at *λ_B_* = 717.0 nm for Red_a mutant and WT PSI, respectively. Reprinted with permission from Ref. [63] Copyright 2020 American Chemical Society.

Figure 10A compares two resonant HB spectra (curves b and e; *λ_B_* = 707.7 nm) obtained for the Red_a mutant and WT PSI, respectively, from *Synechocystis* PCC 6803. A comparison reveals that for this excitation, the EET to the lowest C714 trap is present, as indicated by the shoulder near 714 nm labeled by the thick blue arrow next to curve e. The C714 trap is even more clearly observed in the spectral hole (Figure 10A, curve f) obtained using non-resonant excitation at 670.0 nm. This is consistent with our earlier studies of single WT PSI complexes [66], where we showed that the C706/C707 (lumped together into one state in [66]) and C714 states are connected by fast EET, ~5 ps. However, in the Red_a mutant, this pathway is significantly disrupted due to the C706 state being shifted to 710 nm (becoming the C710 state). The latter can then be bleached via non-resonant excitation (see asterisk in spectrum c). Note that the new C710 state emits independently, i.e., it does not transfer energy to the C714 trap, while the C707 state still efficiently transfers energy to C714. We will show below that both C710 and C714 fluoresce independently, at 725 nm and 722 nm, respectively.

**Figure 11:**
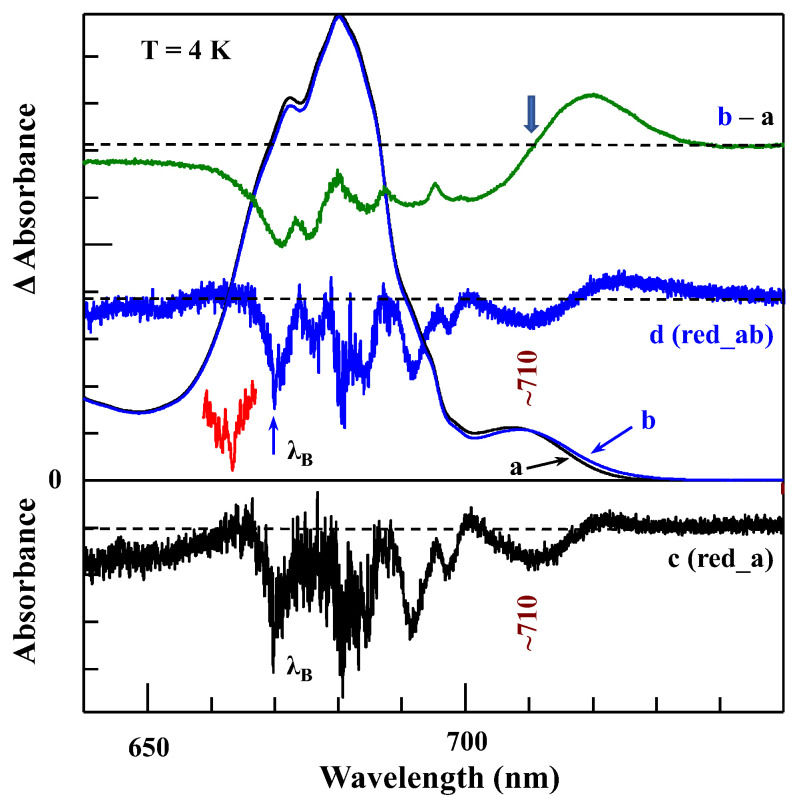
Spectra a and b correspond to absorption spectra of the Red_a and Red_ab mutants. Curve (b–a) is the difference between spectra b and a. Low fluence (*f* = 135 J/cm^2^) HB spectra of Red_a and Red_ab are shown as curves c and d, respectively; *λ_B_* = 670.0 nm; *T* = 4 K. Red insert hole (below curve d) was obtained with *λ_B_* = 665.0 nm. *T* = 4 K. Reprinted with permission from Ref. [63] Copyright 2020 American Chemical Society.

*Comparison of various optical spectra obtained for Red_a and Red_ab mutants.* Spectra a and b in Figure 11 are the absorption spectra of the Red_a and Red_ab mutants, respectively, normalized for pigment content. Their difference spectrum, b–a (multiplied by a factor of 10) is shown as a green curve, which clearly reveals a red shift of some absorption from about 707 nm to about 716 nm. Replacement of His95 in PsaB with Gln95 (Red_b mutant) alone also induces a similar spectral shift, as well as changes across the 650–700 nm region. We refer to this new/shifted C707 state as C716 (see Table 1), and the shift is clearly attributed to the mutation from B7–A31–A32-Hist 94 to B7–A31–A32-Gln. The most red-shifted state in the Red_ab mutant is also assigned to the B7–A32–A31-Gln cluster. The observed C707 → C716 red shift is likely due to increased excitonic couplings within the B7–A32–A31-Gln cluster. Spectrum d for the Red_ab mutant in Figure 11 suggests that energy transfer from C716 → C714 may occur only for a relatively small subset of complexes where C716 ZPL is still higher in energy than the C714 ZPL. Therefore, the remaining fraction of C716 ZPLs will fluoresce, leading to a significantly red-shifted emission band with a maximum near 735 nm [63]. As mentioned above, the C710 (shifted C706) does not transfer energy to the original C714 lowest-energy trap in both the Red_a and Red_ab mutants. This state emits at ~725 nm (see Table 2 and Ref. [63]). We conclude that the protein scaffolding plays an important role in EET dynamics, and it can be altered even by single- and double-point mutations. 

Spectra c and d in Figure 11 correspond to the non-resonant HB spectra obtained for Red_a and Red_ab mutants, respectively, with *λ_B_* = 670.0 nm, *T* = 4 K. The red curve below curve d (double mutant) is an enlarged ZPH burned at 665.0 nm, featuring a fwhm of ~8 cm^−1^. A similar width of the ZPH at the same *λ_B_* was obtained for the Red_a mutant. Such ZPH width corresponds to a relaxation time of 1.3 ps. This ZPH appears to be superimposed on an even broader hole, reflecting sub-ps EET in agreement with the 2DES data [5]. We note that the low-energy bleaches in curves c and d (Red_a and Red_ab) are peaked at 710 nm and mostly reflect a bleach of the new C710 state (original C706 state shifted by addition of B33 Chl). Both spectra c and d in Figure 11 show red-shifted antiholes (vide supra); however, the additional blue-shifted antiholes are not clearly resolved for λ_B_ = 670.0 nm due to overlap with the bleached higher-energy states. 

**Figure 12:**
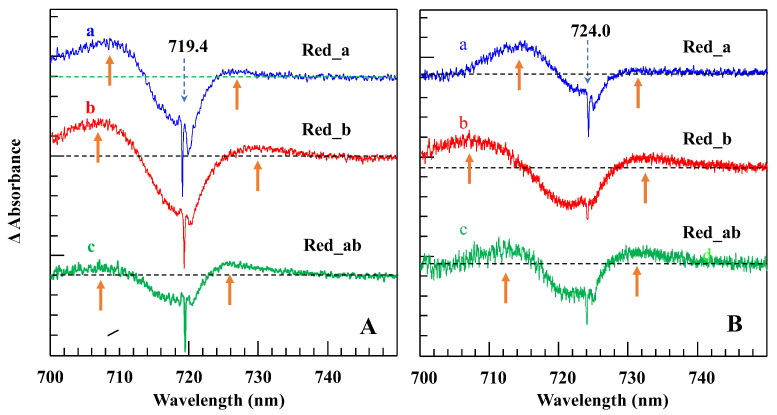
(**A**). Resonant HB spectra obtained for the Red_a (curves a), Red_b (curves b), and Red_ab (curves c) mutants obtained at excitation wavelengths of 719.4 nm. (**B**). The same for 724.0 nm. Upwards arrows point to the maxima of blue- and red-shifted antiholes. Reprinted with permission from Ref. [63] Copyright 2020 American Chemical Society.

*Comparison of resonant holes obtained for the Red_a, Red_b, and Red_ab mutants.* Figure 12 compares resonant holes obtained for the three mutants at two different burning wavelengths (i.e., 719.4 nm and 724.0 nm in frames A and B, respectively). Very intense real PSB holes revealed in the HB spectra of Figure 12 indicate strong el-ph coupling (*S*), consistent with the red-shifted emission spectra analyzed below. The Stokes shift values can be estimated from Table 2. Since theoretical modeling of resonant HB spectra is beyond the scope of this review, here, we only mention that the overlap between the real- and pseudo-PSBs on one hand, and differently shifted antiholes (see Figure 1) on the other hand (due to the mutation-modified energy landscape) will have to be taken into account in a theoretical description of the resonant holes (research in progress). Qualitatively, the mutations above affect the shapes and maxima of both blue- and red-shifted antiholes. The latter indicates that mutation not only modifies trap energies and EET dynamics, but also changes the protein energy landscape around the molecules contributing to a particular trap (research in progress). The red-shifted antiholes are not observed in WT PSI (vide supra). The blue-shifted antiholes are typically observed in HB spectra since the molecules that shift red upon burning can still be burned via the PSB, albeit with much smaller probability. When burning occurs via the PSB, molecules whose transitions were shifted to the red may return to the original transition frequency (for simple TLS model, Figure 1), or their transition frequencies can be shifted further blue or red depending on the complexity of the protein energy landscape, which can be multidimensional and multi-well. The energy landscape is responsible for the asymmetry observed in the spectra in Figure 12, which can be interpreted as an “effective” NPHB yield being higher for blue shifts of the transition energies.

Table 1 summarizes the most recent assignments of the low-energy states of PSI [63]. The first four columns correspond to WT PSI, Red_a, Red_b, and Red_ab mutants of *Synechocystis* PCC 6803. The fifth column lists the lowest-energy states reported previously in the literature for *T. elongatus* [66,67,71,86,98,142,143,144,145], along with the assignments guided by data obtained for WT *Synechocystis* PCC 6803 and its three mutants [63]. In Table 2, we present the connections between the absorption bands discussed above and the emission bands.

**Table 1:** Lowest-energy states in WT PSI from *Synechocystis* PCC 6803 and its three mutants, according to Ref. [63]. The maxima of low-energy states for *T. elongatus* are in part adopted from [66,71], though Chl composition of the C710 and C719 states of *T. elongatus* has been assigned based on data from [63,67].

WT *Synechocystis*	Red_a Mutant *Synechocystis*	Red_b Mutant *Synechocystis*	Red_ab Mutant *Synechocystis*	WT T. elongatus (from Refs [63,67])
C706 *^a^* (B31-B32)	C710 *^b^* (B31-B32-B33)	C706 *^a^* (B31-B32)	C710 *^b^* (B31-B32-B33)	C710 *^b^* (B31-B32-B33)
C714 ^c^ (B37-B38)	C714 *^c^* (B37-B38)	C714 *^c^* (B37-B38)	C714 *^c^* (B37-B38)	C715 *^d^* (B37-B38)
C707 *^a^* (His95-B7-A31-A32)	C707 *^a^* (His95-B7-A31-A32)	C716 *^e^* (Gln 95-B7–A31–A32)	C716 *^f^* (Gln 95-B7–A31–A32)	C719 *^g^* (Gln94-B7–A31–A32)

^(*a*)^ In agreement with our previous assignment for bulk and single PSI complexes of *Synechocystis* PCC 6803 [66]. Our analysis suggests that there are three major red states in *Synechocystis* PCC 6803, with C706 and C707 states nearly degenerate in WT PSI. The Red_a mutant affects the C706 state, while the Red_b mutation modifies the C707 state. ^(*b*)^ The mutation-induced spectral shift from 706 to 710 nm (C706 → C710). This trimer is also present in *T. elongatus* and is assigned to the C710 state. However, BChl B33 in *T. elongatus* has a slightly different orientation: its porphyrin plane is rotated by ~10° relative to that of the Red_a mutant. Thus, the C710 in *T. elongatus* is not identical to that observed in the Red_a mutant. ^(*c*)^ In agreement with our previous assignments; see [62,69]. According to [67], the lowest excitonic state of the B37–B38 dimer has to be the weaker one. ^(*d*)^ We assign the C715 in *T. elongatus* to the B37–B38 dimer, by analogy to the C714 trap observed in *Synechocystis* PCC 6803, although in [144,147], the C715 state in *T. elongatus* was assigned to the B7–A32–A31 cluster and in [86,87], it was assigned to the B31–B32–B33 trimer. ^(*e*)^ Revealed by a comparison of WT and Red_b mutant, so the Red_a mutation does not affect the energy of the C707 state (see main text). ^(*f*)^ Revealed by a comparison of the Red_a and Red_ab mutants. ^(*g*)^ See [67], though other assignments were also proposed; see [71,86,87,142,143,144]. The C719 trap in *T. elongatus* emits around 740 nm [67,142] (similar to the F735 emission originating from the C716 state in the Red_ab mutant of *Synechocystis* PCC 6803; see Table 2). Importantly, no sharp emission lines were observed for this state in single PSI complexes of *T. elongatus*, suggesting that el-ph coupling is also strong in these PSI complexes [66,142].

**Table 2:** Assignment of emission bands originating from the low-energy absorption states in WT *Synechocystis* PCC 6803 and its three mutants.

WT PSI and Mutant *Synechocystis*	Low-Energy Emitting States/Stokes Shift (cm^−1^)	Assignment of Fluorescence Bands
WT	C706/119 C707 C714/150	F712 (minor) *^b^* F722 (major)
Red_b mutant	C706/119 C714/144 C716 *^a^*	F712 (minor) F722 (major) F735 (<1%)
Red_a mutant	C714/150 C710 *^c^*/292	F722 (~53%) F725 (~47%)
Red_ab mutant	C710/292 C714/150 C716 *^a^*	F725 (~80%) F722 (~12%) F735 (~8%)

^(*a*)^ Stokes shift cannot be estimated due to a very weak and broad emission spectrum; recall that the C716 state is the shifted C707 state present in WT PSI and is present in both Red_b and Red_ab mutants due to mutation from His95-B7–A31–A32 to Gln 95-B7–A31–A32. ^(*b*)^ No emission from the C707 state in WT PSI as the C707 transfers energy to the C714 state. ^(*c*)^ The C710 state in *Synechocystis* PCC 6803 is present in the Red_a and Red_ab mutants since Chl B33 was added to the B31–B32 dimer (vide supra).

The large values of Huang–Rhys factors (*S*), in a 3 to 5 range, depending on the red state and red-shifted emission spectra suggest that red states possess a strong contribution from Chl-Chl CT intra-dimer states. It is well known that such states steal (borrow) oscillator strength via electronic coupling with the parent exciton state(s) [166,167]. Due to strong mixing with the CT states, the emission bands are typically broad due to large homogeneous bandwidth. This is not specific to cyanobacterial PSI since fluorescence spectra in many photosynthetic antennas are also largely contributed to by the CT states [167,168,169]. For example, the emission spectra of aggregated LHCII complexes were also significantly red-shifted [168], and resonant holes burned in the low-energy wing of their absorption spectrum (685–710 nm) showed high el-ph coupling strength with *S* factors of 3–4 [169]. Weak ZPHs and large contributions from the real PSBs observed in Figure 12 are consistent with CT character of the emission spectra obtained for WT PSI and its three mutants. The findings of Ref. [63] show that, in particular, the C710 state (of the *Synechocystis* PCC 6803 Red_a mutant) is characterized by a large *S* factor (about 5), while the original C706 and C714 show smaller Stokes shifts (i.e., smaller *S* values). Unfortunately, exact el-ph coupling strengths cannot be reliably determined from resonant holes burned into a superposition of more than one overlapping low-energy state. This could be addressed in future studies via theoretical modeling of the resonant holes using the HB master equation [34,97,101,102,104] and/or non-Markovian density matrix approach [57,72]. The strong el-ph coupling reported above correlates well with large permanent dipole moment changes (*f*∆*μ* ~ 2.3 D) and the large pressure-induced shift rates of spectral holes (R_p_ ~ 0.5 cm^−1^/MPa) observed for the C714 state in WT PSI of *Synechocystis* PCC 6803 [65,69]. 

In summary, in this section, we demonstrated that upon addition of the B33 Chl the C706 state in the WT PSI of *Synechocystis*, PCC 6803 (originally due to the B31–B32 dimer) shifts to 710.4 nm, forming the C710 state, which emits near 725 nm (F725 band; see Table 2). A similar state seems to be also present in *T. elongatus* (see Table 1). Narrow lines observed in the SPCS spectra of *T. Elongatus* PSI at around 710–712 nm [66,86] may belong to C715 or the newly proposed (see below) C712 [67]. Replacement of His95 in PsaB with Gln95 results in a shift of the C707 state to 716 nm. As shown above, there is an efficient EET from the original C707 state to the lowest energy trap (C714) in WT and Red_b mutant, but it is less efficient in the Red_a and Red_ab mutants. This suggests that these mutations modify both the EET pathway(s) and protein energy landscape (reflected by differently shifted and shaped antiholes). Importantly, we find that the C716 state in the Red_b and Red_ab mutants is similar to the C719 trap observed in *T. elongatus* [66,69,86,87,170]. This, in turn, suggests that the lowest-energy state in *T. elongatus*, i.e., the C719 state*,* could be also assigned to the Gln94-B7–A31–A32 cluster located on the luminal side. This disagrees with Ref. [144], where the lowest lying state in *T. elongatus* was assigned to the trimer B31–B32–B33, followed by the B7–A32–A31 trimer. However, based on our data (see Table 1) and discussion in Section 3, the B31–B32–B33 trimer definitely contributes to the C710 state in Red_a and Red_ab mutants. The B31–B32–B33 trimer was also assigned to the C710 state in *T. elongatus* in [71]. Recently, it was reported that the Chl *a* cluster mostly affected by switching between trimeric and monomeric PSI of *Synechocystis* PCC 6803 is B7–A32 [11]. This is in agreement with the assignments above since the difference between monomer and trimer absorption spectra for *Synechocystis* PCC 6803 was maximal at around 707 nm rather than 714 nm [65]. Thus, both cyanobacteria possess the lowest energy trap near P700 to ensure efficient EET to the RC, as red traps serve to focus energy into P700 [11,69,142,152,170]. The C707 state (His95-B7–A32–A31) in WT PSI is not the lowest-energy trap in *Synechocystis* PCC 6803*,* most likely due to smaller coupling constants between Chls of this cluster in comparison with the PSI of *T. elongatus*. Finally, if one assumes that B37–B38 is responsible for one of the red states in *Synechocystis* PCC 6803 and *T. elongatus* (given the evidence presented in [66,67,142] and reviewed in Section 3) in light of the information above, in *Synechocystis* PCC 6803, it must be the C714 state.

**Figure 13:**
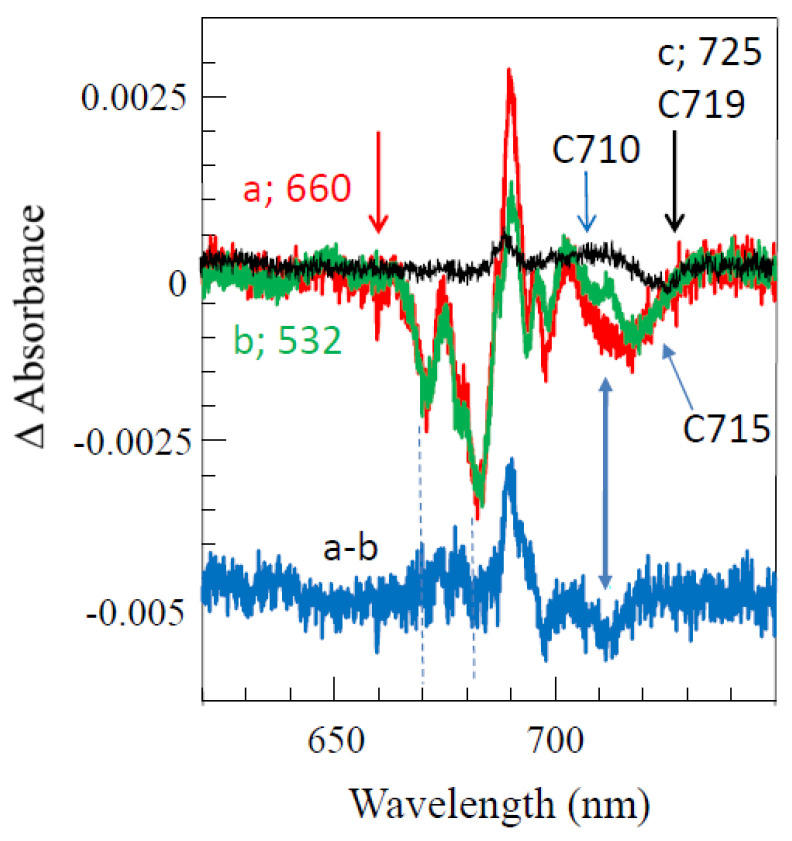
PSI of *T. elongatus* with oxidized P700 (emission peak at 732 nm). Hole spectra for *λ_B_* of 660 nm (red, a, resonant hole is somewhat visible), 532 nm (green, b), and 725 nm (black, c). Blue a–b: the difference between curves a and b, the hole spectrum of suspected C712. Holes due to C710, C715, and C719 states are also shown. Adopted in modified form from Ref. [67] Copyright 2016 American Chemical Society.

As briefly mentioned above, preliminary HB evidence exists for yet another pool of red Chls in PSI of *T. elongatus.* Figure 13 depicts persistent non-resonant hole spectra from [67] produced by illumination at 660 nm (red) and 532 nm (green, excitation via carotenoids [154]). These hole spectra are normalized at the deepest non-resonant satellite holes at 672 and 683 nm, as well as the red-most edge of the hole spectrum past 719 nm. The black curve represents the hole spectrum for 725 nm illumination, selectively probing the C719 state. The hole burning yield is poor, as the lifetime of the C719 state is shortened due to quenching by P700^+^ [142]. Note that the lowest-energy feature of the black curve is the most intense one, indicating that C719 belongs to the Chl cluster with the lowest excitonic state being enhanced by borrowing oscillator strength, again, likely B7–A32 (+B6 + A31), in agreement with analysis given in Ref. [63]. The long-wavelength region of the green curve is dominated by sufficiently well-resolved holes at 715 and 710 nm, corresponding to the C715 and C710 states. The green spectrum is similar to those reported in [71]. Thus, the 712 nm hole in the blue, a–b, spectrum appears distinct from either C710 or C715. The respective Chls are unlikely to be reached via EET starting from the carotenoids.

### 4.2. Possible Energy Transfer Pathways in PSI Complex, PSI Mutants, and Resulting Fluorescence Maxima

**Figure 14:**
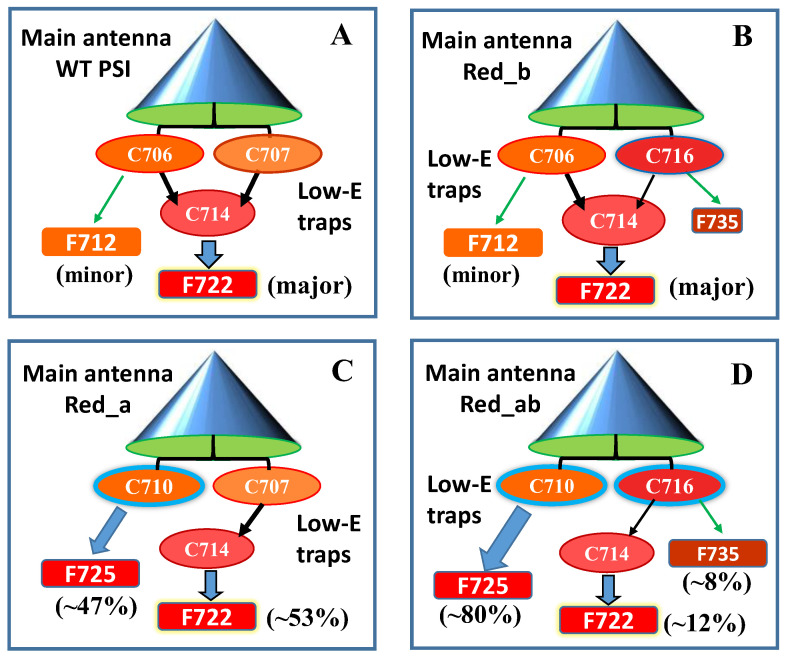
Schematic representation of simple models illustrating possible EET pathways and resulting fluorescence (F) maxima in WT PSI (**A**), Red_b (**B**), Red_a (**C**), and Red_ab (**D**) mutants of *Synechocystis* PCC 6803. Black arrows indicate downhill EET from the main antenna to the low-energy states. Thin green and thick blue arrows point to the resulting minor and major emission band maxima, respectively. Reprinted with permission from Ref. [63] Copyright 2020 American Chemical Society.

The discussion above is summarized in Figure 14. It indicates that in the Red_a and Red_ab mutants, there is most likely no EET from C710 (C706 in WT) to the C714 trap (see frames C and D), as indicated by the strong F725 emission bands. In contrast, C714 is efficiently populated in the WT PSI and Red_b mutant, as illustrated in frames A and B. The very weak emission near 712 nm in WT and Red_b is likely observed due to a very broad distribution of EET rates, i.e., for a fraction of complexes, the radiative decays could compete with the EET rates. The emission from the C707 state in WT and Red_a mutant is suppressed, in agreement with HB spectra shown in Figure 10A, due to efficient EET from the C707 state to the emitting C714 trap. 

Recall that His95 mutation to Gln shifts the C707 state to C716. As a result, only the ZPLs (within the C716 band) whose energies are higher than ZPLs within the C714 band can transfer energy to the C714 trap. Therefore, the ZPLs whose energies are lower than the ZPLs of the C714 band will emit at T = 4 K, as clearly observed for the Red_ab mutant (see F735 band). This most likely occurs because the contribution from the F722 band is relatively weak. Note that excitation trapped in the C710 state in the Red_a and Red_ab mutants does not reach the C714 state and emits at 725 nm (F725), in agreement with the HB spectra.

### 4.3. Persistent Holes Associated with Primary Charge Separation in the PSI_3_ RC with Low-Energy Excitation

**Figure 15:**
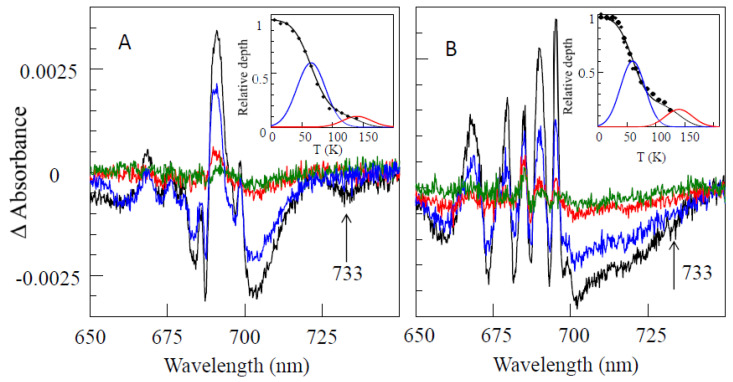
Thermocycling effects on the P700^+^ minus P700 difference spectrum resulting from 733 nm illumination in chemically reduced samples (**A**) *T. elongatus* and (**B**) *Synechocystis*. PCC 6803. Hole spectra right after burning (black), after thermocycling to 55 K (raising temperature to 55 K for one minute and lowering it back to 5 K, blue), 105 K (red), and 139 K (*T. elongatus*) or 125 K (S*ynechocystis* PCC 6803) (green). Inserts depict the distributions of the barriers deduced from the recovery of the main bleach at 703 nm. Two separate components of the distribution are depicted with blue and red curves. NPHB hole at 733 nm can also be seen. Adopted in modified form from Ref. [67] Copyright 2016 American Chemical Society.

It was noticed some time ago that absorption, electron paramagnetic resonance (EPR), and magnetic CD (MCD) signals in PSI extend beyond 800 nm [3,138,171,172] and that excitation at these wavelengths could still result in charge separation [138,171,172] in some plants and cyanobacteria. Schlodder et al. have shown that the fluorescence of the red-most antenna state in *T. elongatus*, C719/F740 can be suppressed due to the effective downhill EET to the oxidized P700 (P700^+^) [142]. This indicates that the respective cluster of antenna Chls is most likely located relatively close to the P700 special pair (excluding the B31–B32–B33 trimer as a likely candidate for C719/F740 in *T. elongatus*). As mentioned above, B33 is not present in WT *Synechocystis* PCC 6803, and only B31–B32 dimer is present [108,118,119]. In T. elongatus, the peak of the emission band shifts by almost 10 nm depending on the redox state of P700 [142]. When P700 is oxidized, the lowest C719/F740 state is quenched due to EET to P700^+^, its lifetime is shortened, and the emission and the NPHB spectra become dominated by the second-lowest C715 state, emitting at 732 nm. (This also indicates that C715 and C719 are competing traps at low temperatures.) P700^+^ absorbs at lower energies than the C719/F740 state and energy is easily transferred from this state to P700^+^. It was demonstrated that excitation at wavelengths as long as 800 nm results in charge separation at cryogenic temperatures also in WT *Synechocystis* PCC 6803 [67]. Fluorescence of the other red states appears less sensitive to the redox state of P700 [67,142]. These results support the assignment of the lowest state in *T. elongatus* to the B7–A31–A32 Chl cluster. One may note that charge separation in PSI at cryogenic temperatures results in persistent and not transient spectral holes, which are, on the other hand, photochemical in nature but reversible. The yield of charge separation is extremely high (almost one) and in order to observe this kind of persistent P700 hole, one needs to perform experiments in complete darkness, and start with samples with P700 in a reduced state (FT spectrometers are not suitable for this type of experiment). Figure 15 depicts such P700+ minus P700 holes, obtained with illumination at 733 nm, as well as demonstrates that an increase in temperature is required for hole recovery. Similar P700 holes of smaller amplitude can be obtained with up to 800 nm illumination. Additional derivative-like features observed in the hole spectra at higher energies are most likely due to electrochromic shifts of the antenna pigments located close enough to the electron transfer chain. A similar technique involving temporary increases in temperature (thermocycling) [97,104] can be used to separate different contributions to persistent resonant and non-resonant non-photochemical holes since protein energy landscape parameters may vary from one Chl environment to another, and some overlapping holes may recover faster than others.

## 5. Applications of Frequency Domain Methodologies to PSI_3_–IsiA_18_ Supercomplexes and IsiA Monomers

**Figure 16:**
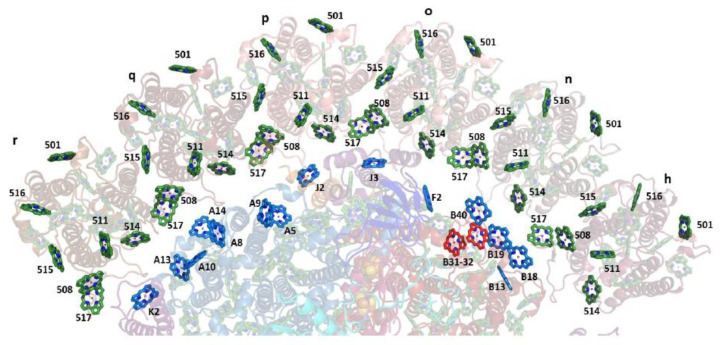
Structure of the IsiA hexamer; Chls likely contributing to the lowest-energy state(s) in IsiA monomers are shown in green. A part of the PSI core monomer is also shown, with core Chls at the interface between the IsiA ring and the PSI monomer highlighted in blue. The B31–B32 dimers are highlighted in red. The supercomplex is based on WT *Synechocystis* PCC 6803 PSI; therefore, this dimer is likely the C706 low-energy trap [63]. (B33 is not present in supercomplexes of WT *Synechocystis* PCC 6803). Chl labeling is according to [64]. Reprinted with permission from Ref. [64] Copyright 2022 American Chemical Society.

As stated above, iron-deficient conditions result in the formation of PSI_3_–IsiA_18_ supercomplexes [120,121,122,123,124,125]. Figure 16 shows the structure of six neighboring IsiA monomers—r, q, p, o, n, and h–and labels several pigments that could contribute to the lowest-energy state(s) of the IsiA monomers. 

Over the years, many emission and absorption spectra for the PSI_3_ core, PSI_3_–IsiA_18_ supercomplexes, and isolated IsiA complexes have been reported [62,63,64,68,158]. Unfortunately, low-temperature optical spectra vary somewhat from one study to another, complicating modeling. In particular, emissions associated with the IsiA ring feature somewhat varying peak wavelengths, suggesting modified emitting states of IsiA antennae. Ref. [158] attributed the observed 685 nm → 682 nm shift in the emission spectra upon switching from the intact ring to a collection of non-interacting IsiA monomers to modified pigment–pigment interactions between the IsiA monomers and a lack of EET among the IsiA monomers and/or to modified interactions between the ring and PSI_3_ trimer in the PSI_3_–IsiA_18_ supercomplex. However, there were also differences in the absorption and emission spectra of isolated IsiA monomers [62,64,68,158], which might be due to different number of Chls per monomer, as some Chls could be lost during isolation/purification procedures. The latter could lead to different pigment composition of the lowest-energy excitonic states and to shifted emission maxima. Moreover, it was often impossible to determine, in retrospect, whether P700 in PSI supercomplexes or PSI_3_ trimers reported in various earlier works were in an oxidized or partially reduced state (see Section 4.3). We focus below on very recently reported data from our laboratory, where a lot of effort was put into keeping the P700 in a well-defined (oxidized) [67,142] state. We present the modeling of various optical spectra and discuss the EET processes, as well as some hole burning results.

### 5.1. Comparison of Low-Temperature Emission and Absorption Spectra for Isolated IsiA Monomers, PSI_3_ Trimer, and PSI_3_–IsiA_18_ Supercomplex

**Figure 17:**
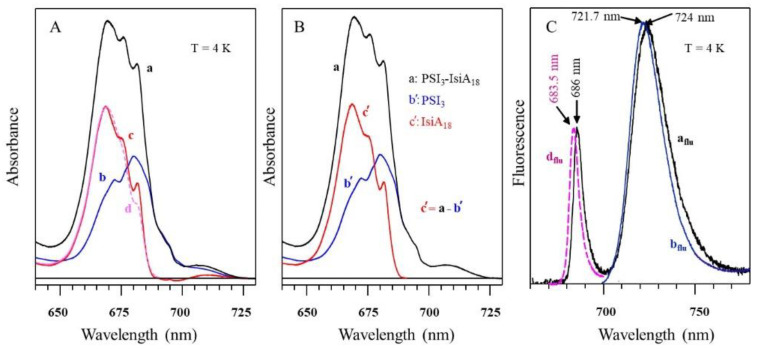
(**A**). Curves a, b, and d are measured 4 K absorption spectra of the PSI_3_–IsiA_18_ supercomplex, the isolated PSI_3_ trimer, and the isolated IsiA monomer, respectively. Absorptions of the PSI_3_–IsiA_18_ supercomplex and the isolated PSI_3_ are area-normalized according to Chl content. Curve c (red) is the difference between spectra a and b. (**B**). Spectra c′ and b′ correspond to corrected (“pure”) contributions assigned to PSI_3_ trimer and IsiA_18_ ring, respectively; see text for details. Curve a is the same as in frame (**A**). (**C**). Emission spectra of the PSI_3_–IsiA_18_ supercomplex (a_flu_), the isolated PSI_3_ trimer (b_flu_), and the isolated IsiA monomer (d_flu_). Spectra are normalized to emissions maxima. Reprinted with permission from Ref. [64] Copyright 2022 American Chemical Society.

Curves a and b in Frame A of Figure 17 are 4 K absorption spectra of the *Synechocystis* PCC 6803 PSI_3_–IsiA_18_ supercomplex (591 Chls) and isolated PSI_3_ trimer (285 Chls), respectively, normalized for the expected pigment content. The corresponding emission spectra (labeled as a_flu_ and b_flu_ and normalized to emissions maxima) are shown in Frame C. The absorption spectrum of the PSI_3_–IsiA_18_ supercomplex shows three maxima located near 670, 676, and 682 nm, as well as an additional weak band near 710 nm. Spectrum c (red) in frame A is the difference between spectra a and b assigned mostly to the absorption of the IsiA_18_ ring. Note that IsiA_18_ absorption spectrum (c = a − b) contains a pronounced peak near 683 nm, resembling the narrow peak of CP43 core antenna complex of PSII [54], as well as a weak band at 710 nm. The latter band must belong to the PSI_3_ core. If it belonged to the IsiA ring absorption, this would mean that each IsiA monomer must have a red-shifted trap, which is inconsistent with the significant amount of the ring emission peaked at 686 nm and fast intra-monomer EET. Therefore, the comparison of spectra a, b, and c suggests that PSI_3_ core residing within the supercomplex features a somewhat larger oscillator strength of the low-energy trap(s). This is likely due to small structural differences between PSI_3_ cores in samples grown under different conditions, leading to a weaker oscillator strength of the low-energy trap(s) and blue-shifted and broader PSI_3_ emission (the maxima of spectra a_flu_ and b_flu_ are at 721.7 and 724.0 nm, respectively; frame C). As shown in Ref. [64], the integrated intensity of curve c corresponds to about 309 Chls, i.e., 18 IsiA monomers with about 17.17 Chls per a single IsiA. Section 5.3 and Section 5.4 summarize the modeling results from Ref. [64].

Curve c (red) in frame B of Figure 17 was corrected by removing the long-wavelength contributions assigned to PSI_3_, as there is no reasonable basis for “red states” in the IsiA_18_ ring. Thus, the corrected curve (c′ in frame B) in a good approximation can be assigned to the absorption of the intact IsiA_18_ ring within the ring–core supercomplex. Taking into account this correction, the sample studied by Reinot et al. [64] contained about 17 Chls per IsiA monomer within the IsiA_18_ ring, in very good agreement with [124,159]. Thus, structure [124] was used in the ring modeling studies discussed in Section 5.4 below. We emphasize that the absorption spectrum (curve d in frame A; dashed line) obtained for isolated IsiA monomers, in this case, normalized to the maximum of curve c, is similar in shape to the spectrum c. However, its lower integrated intensity suggests that some Chls went missing upon isolation and purification of the IsiA monomers. As a result of both isolation per se (removing inter-IsiA interactions) and the possible loss of pigments, the fluorescence spectrum of the isolated IsiA monomers (d_flu_) was blue-shifted to 683.5 nm, compared with the 686.0 nm emission maximum observed for the IsiA_18_ ring being part of the PSI supercomplex. Overall, it can be estimated that about 2.6–2.7 Chls have been lost in isolated IsiA monomers. The latter was consistent with the modeling studies (see Section 5.3 and Section 5.4).

### 5.2. Hole-Burned Spectra

**Figure 18:**
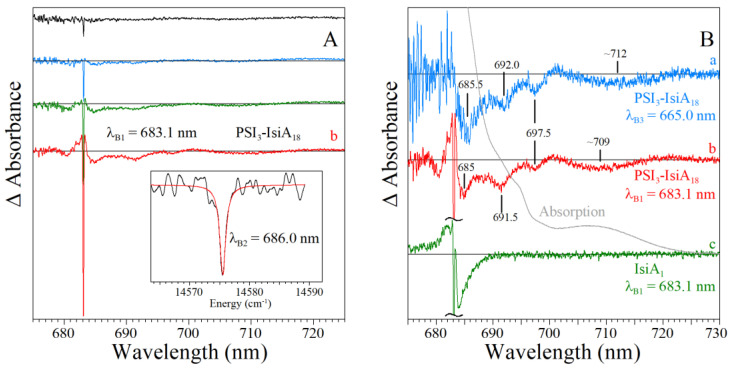
(**A**). Spectra from top to bottom are resonant HB spectra obtained at 5 K for the PSI_3_–IsiA_18_ supercomplex for burning wavelength (λ_B1_) of 683.1 nm (2 cm^−1^ resolution) as a function of illumination dose (fluence). The fluences (from top to bottom) were 3.3, 16.5, 29.7, and 56.1 J/cm^2^, respectively. In this case, the ZPH width is resolution-limited. The inset shows a higher resolution (i.e., 0.5 cm^−1^) ZPH burned at λ_B2_ = 686.0 nm. (**B**). Curves a (blue) and b (red, shown also in red in frame **A**) are HB spectra obtained for the PSI_3_–IsiA_18_ supercomplex with λ_B3_ = 665.0 and λ_B1_ = 683.1 nm, respectively. The green curve in frame (**B**) is the HB spectrum obtained for the isolated IsiA monomers (λ_B1_ = 683.1 nm). The low-energy absorption spectrum of PSI3–IsiA18 (gray curve) is shown for comparison. Reprinted with permission from Ref. [64] Copyright 2022 American Chemical Society.

**Figure 19:**
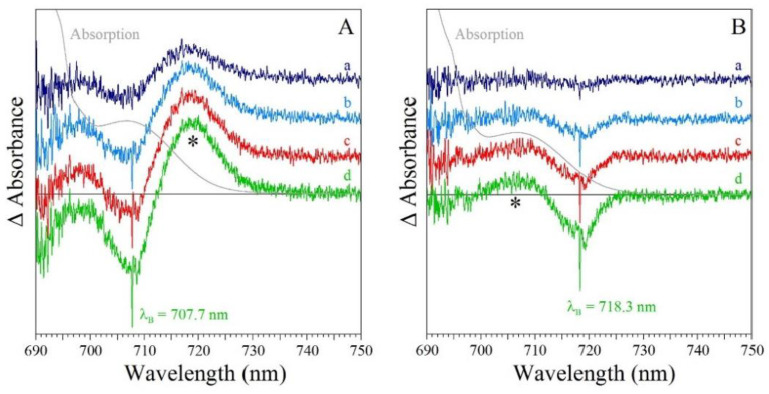
Persistent holes in the red-state region at 5 K. (**A**). Curves a–d were obtained for the PSI_3_–IsiA_18_ supercomplex with *λ_B_* of 707.7 nm. (**B**). HB spectra obtained for λ_B_ = 718.3 nm. Spectra a–d in both frames (**A**) and (**B**) were obtained with an increasing burning fluence of 3.6 (black), 10.8 (blue), 25.2 (red), and 54.0 (green) J/cm^2^, respectively. The asterisks indicate the antihole location. Reprinted in modified form with permission from Ref. [64] Copyright 2022 American Chemical Society.

As mentioned in Section 2.2, the non-resonant HB spectra can reveal the lowest energy trap(s) excited via EET from IsiA_18_ to the PSI_3_ core. Resonant holes, on the other hand, can provide information on el-ph coupling strength (*S*) for low energy pigments. The corresponding fwhm of the ZPHs can provide information on the excited state lifetimes (T_1_). Examples of HB spectra and the information they provide are given in Figure 18 and Figure 19. Figure 18A shows four resonant HB spectra obtained with λ_B1_ = 683.1 nm and 2 cm^−1^ spectral resolution for the PSI_3_–IsiA_18_ supercomplex as a function of burn fluence (illumination dose). Recall that in this case, the ZPHs are resolution-limited. However, the inset in Figure 18A shows higher resolution (0.5 cm^−1^) ZPH burned at λ_B2_ = 686.0 nm with a fwhm of the ZPH of 1.2 cm^−1^. The lowest HB spectrum (red curve b in frame A), with a truncated ZPH, is also shown with more details in frame B (curve b) of Figure 18. 

The T_1_ values are related to the homogeneously broadened line widths, *Γ_hom_*, where *Γ_hom_* = ½ *Γ_ZPH_*. [34,36]. ZPH with λ_B2_ = 686.0 nm and with a fwhm of the ZPH of 1.2 cm^−1^, shown in Figure 18A (corrected for 0.5 cm^−1^ resolution of the FT spectrometer) corresponds to the average T_1_ ~ 11 ps. This is attributed to EET from IsiA to PSI_3_. Note that the 683.1 nm burning in the case of IsiA_1_ monomers (spectrum c in Figure 18B) reveals only a narrow ZPH and corresponding relatively narrow phonon sideband, as downhill EET is not possible (confirming that the state at ~683 nm is the lowest one in the isolated IsiA and justifying correction to the difference of absorption spectra above). For supercomplexes (curve b), the bleaches near 692.0 and 697.5 nm belong to the PSI_3_ trimer, as revealed by a comparison with the HB spectrum obtained at 683.1 nm for the isolated IsiA monomers (curve c). This is also in agreement with the earlier HB spectra burned in the isolated PSI_3_ trimer in [63]. The bleach near 685.5 nm in curve a (Figure 18B) most likely corresponds to the lowest energy state in the IsiA_18_ ring. Due to imperfect EET to the PSI_3_ core, this state emits near 686 nm (see curve a_flu_ in Figure 17C). More HB spectra can be found in Ref. [64]. Here, we only mention that holes burned at *λ_B_* = 718.3 nm in the PSI_3_–IsiA_18_ supercomplex were similar to those observed (for similar burning wavelength) in the isolated WT PSI_3_ trimer [63,64]. This suggests that the Chl cluster responsible for the C714 trap is unaffected by the addition of the IsiA_18_ ring. Coming back to the data shown in Figure 18B, note that the lowest-energy trap for non-resonant excitation at 665.0 nm (see top blue curve in frame B of Figure 18) is at about 712 nm. In the isolated PSI_3_, trimer this state was assigned to the so-called C714 trap of the PSI_3_ core [63].

Frame A in Figure 19 shows four persistent resonant HB spectra (curves a–d) obtained for the PSI_3_–IsiA_18_ supercomplex with *λ_B_* of 707.7 nm. The low-energy part of the absorption spectrum (gray line) is shown for comparison. Interestingly, in contrast to the HB spectra produced with burning at the same wavelength in the PSI_3_ trimer of WT PSI_3_ from *Synechocystis* PCC 6803 and Red_a mutant [63], in frame A the photoproduct is shifted to *lower energies* (see the asterisk in frame A). Note the large red-shifted antihole (see asterisk in frame A). The delayed emergence of the narrow ZPH at 707.7 nm (compare spectra a and b) suggests that at the burn wavelength of 707.7 nm, there are likely two independent bleaches of Chls with weak and very strong el-ph coupling, respectively, with the latter one showing a higher HB yield, probably due to lower barriers on the protein energy landscape. The presence of two nearly degenerate states at this wavelength is in agreement with [63] and Section 4. Frame B in Figure 19 shows the HB spectra obtained at *λ_B_* = 718.3 nm with an increasing burning fluence at *T* = 5 K (see Figure caption). The HB spectra (a–d) in Figure 19B are most likely burned into the lowest energy C714 trap of the PSI_3_ trimer, as they are similar to those observed (for similar burning wavelength) in the isolated WT PSI_3_ trimer [63]. This suggests that the configurational energy landscape of the Chls constituting the C714 trap per se is unaffected by the addition of the IsiA_18_ ring. However, a comparison of the HB spectra shown in Figure 19A,B reveals that energy landscape of Chls absorbing at 707.7 and 718.3 nm varies significantly.

### 5.3. Modeling of Absorption, Emission, Transient Holes, and CD Spectra of IsiA Monomer

**Figure 20:**
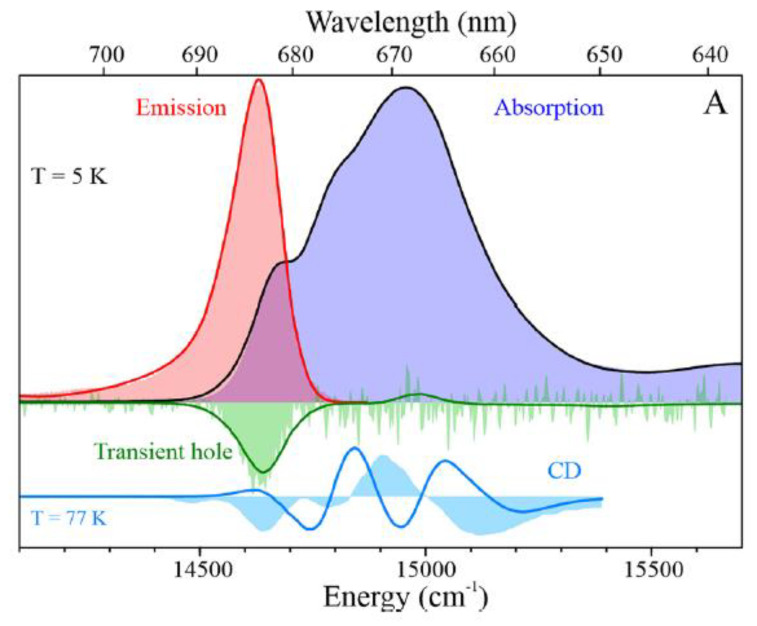

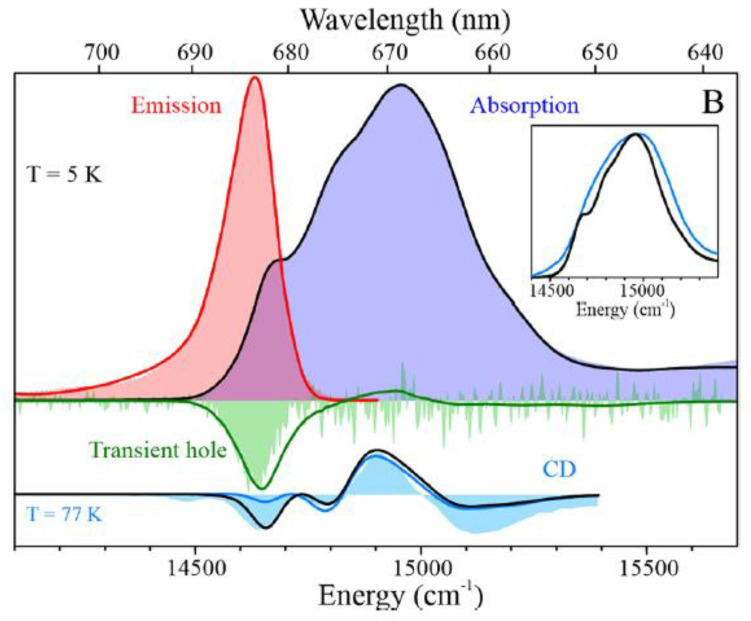
(**A**). Filled curves are experimental absorption (purple), emission (pink), and transient (non-resonant) HB spectra (green) of IsiA monomers at T = 5 K. Emission/HB spectrum were obtained for *λ_ex_*_/_*λ_B_* of 665.0 nm. Solid lines are the best fits to various optical spectra obtained for model *MA*, assuming the absence of Chls 517/501 and 70% loss of Chl 511. Filled lower curve (light blue) and superimposed solid blue line correspond to the experimental and calculated [64] CD spectra at 77 K [158]. (**B**). Filled curves are the same experimental spectra of IsiA monomers as in the left frame. Solid lines are model curves using model *MB*. In this case, the best fits are obtained assuming the absence of Chls 508/517 and 70% loss of Chl 511. The calculated CD spectra, represented by solid blue and black lines, assume 70% loss and 100% occupation of Chl 511. Inset compares 5 K absorption spectrum (black) from [64] with the 5 K spectrum from [158] (blue line). Since the number of Chls likely differed (vide supra), as reflected by somewhat different absorption shapes (see inset in Frame **B**), and the calculated CD curve is based on parameters from fits of 5 K data, whereas the experimental CD was measured at 77 K, no perfect agreement was anticipated. However, the calculated shape is much better in model MB. Reprinted with permission from Ref. [64] Copyright 2022 American Chemical Society.

**Figure 21:**
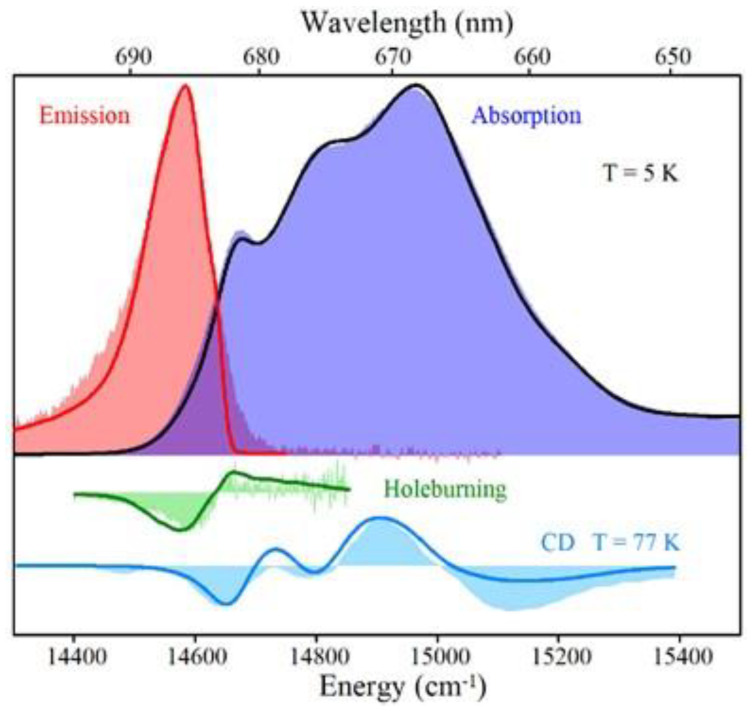
Top three filled curves are 5 K experimental absorption (purple), emission (pink), and non-resonant hole burning spectra of the IsiA_18_ ring. Light-blue filled curve is the 77 K IsiA CD spectrum from [158]. Solid lines represent results of the respective numerical calculation for the entire IsiA_18_ ring. Reprinted with permission from Ref. [64] Copyright 2022 American Chemical Society.

Here, we demonstrate the simulations of various optical spectra obtained for the IsiA monomers (see Figure 20) and IsiA_18_ ring (Figure 21). Filled curves show the experimental absorption (purple), emission (pink), and non-resonant transient HB (green) spectra of the isolated IsiA monomers. The blue-filled spectrum is the CD spectrum from [158]. In the modeling of these spectra [64], the following was assumed: (i) isolated monomers contained the same number of Chls, while the variations in orientations of individual monomers in the IsiA_18_ rings were the same for all rings; (ii) isolated monomers, after possibly losing some Chls, maintained their native coupling coefficients between the remaining pigments; and (iii) protein scaffolding (site energies) also remained unchanged, despite some Chls being lost. The *V_nm_* parameters for individual monomers were calculated using Tr-Esp methodology [51,53,64]. In other words, the experimental spectra of the isolated IsiA monomers were fitted using all 18 sets of *V_nm_* values obtained for the individual monomers of the ring from the cryo-EM structure [124]. The solid lines in Figure 20 are the best fits to the experimental data. In frame A, all the solid lines were obtained using coupling constants calculated for monomer “n”. The best fits of absorption, emission, and HB spectra were obtained assuming that isolated IsiA monomers no longer possess Chls 501 and 517, while Chl 511 is present in only 30% of IsiA monomers (model *MA*). However, the set of parameters obtained in model *MA* cannot provide a good match to the CD spectrum. The filled light-blue curve and the superimposed solid blue line correspond to the experimental [158] and calculated 77 K CD spectrum. Frame B shows another fit of the same experimental data (model *MB*), which can also reasonably describe the CD spectrum. Here, the best fits are obtained assuming the absence of Chls 508 and 517 and a 70% loss of Chl 511. The calculated CD spectrum represented by the solid blue line also assumes that Chl 511 is present in 30% of IsiA, while the black calculated CD curve assumes 100% presence of Chl 511. Thus, it appears that the sample studied in Ref. [158] had its Chls 511 fully intact. None of the other combinations of missing Chls could simultaneously describe all the experimental data shown in Figure 20. Note that model *MB* is feasible as missing Chls 508 and 517 are located close to the interface between the ring’s monomers and could be relatively easily lost upon isolation of the IsiA complexes. All the fits in Figure 20 were obtained with the phonon Huang–Rhys factor *S* = 0.55 (based on the HB data), and the sum of the Huang–Rhys factors for Chl intramolecular vibrations was *S* = 0.3. The exciton energies and their standard deviations averaged over all 18 monomers can be found in the Supporting Information of Ref. [64]. The optical spectra are modeled using a second-order non-Markovian theory [45] with the Nelder–Mead simplex algorithm [173] for parameter optimization. The equations employed in this modeling can be found in the Supporting Information of Ref. [57].

### 5.4. Modeling of Absorption, Emission, Transient Holes, and CD Spectra of the IsiA_18_ Ring

The calculated spectra (solid lines) for the entire IsiA_18_ ring (18 monomers) are shown in Figure 21. The top three filled curves are 5 K experimental absorption (purple), emission (pink), and non-resonant persistent hole-burning of the IsiA_18_ ring. The light-blue filled curve is the 77 K CD spectrum from [158]. Exciton energies and their standard deviations averaged over all the monomers within the ring are shown in Table S10 in the SI of Ref. [64]. Calculations were made for 306 pigments (200k iterations) using *HexB* model for all three hexamers (this is a hexamer model corresponding to the *MB* model above). All the site-energies, inhomogeneities, *S*-factors, and spectral densities, as well as couplings between preserved Chls were included into the model without any change from the respective *MB* model.

Only the emission calculation differed for the hexamer model because the emission spectrum does not just scale up if one adds monomers. In a simplified approach where one naively calculates the lowest state of the aggregate of many IsiA monomers and assumes perfect EET from all other states (i.e., assumes that relaxation is several orders of magnitude faster than the radiative lifetime), emission occurs only from the very lowest-energy exciton state. Such an idealized excitonic emission spectrum of the full IsiA_18_ ring would be further red-shifted and much narrower than the experimentally observed ring emission. For the full intact IsiA_18_ ring, the assumption of full excitonic relaxation to the very lowest state is clearly invalid, as due to site energy disorder, the EET may or may not be possible between the lowest states of the 18 individual IsiA monomers. As argued in Ref. [64], EET between monomers is much slower than intra-monomer exciton relaxation, i.e., it occurs via Förster mechanism, and most likely not more than 2–3 inter-monomer crossings occur before either emission or EET to the core in the intact supercomplex. Thus, one needs to allow some emission from the lowest exciton states of several individual IsiA monomers. See [64] for details.

### 5.5. Most Likely Scenario of EET from the IsiA_18_ Ring to the PSI_3_ Core: Major Core Entry Points

**Figure 22:**
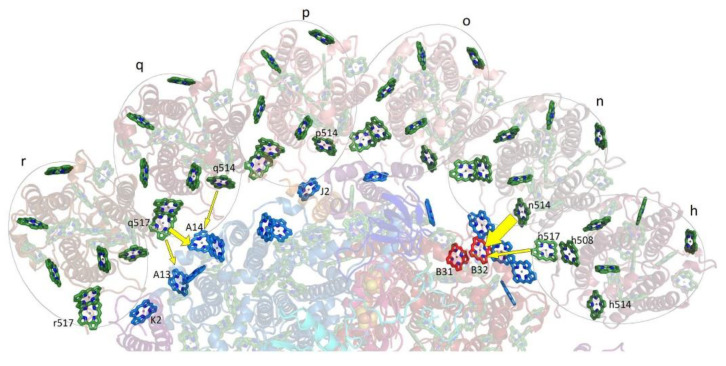
Possible major entry points from the hexamer to the PSI monomer in model *HexB*. The six IsiA monomers are the same as in Figure 16. Chls in blue and red belong to the PSI core. Chls in green belong to the IsiA ring. Arrows indicate likely entry points for excitation from the ring to PSI monomer. Labeling of Chls according to [64]. Reprinted with permission from Ref. [64] Copyright 2022 American Chemical Society.

Further progress can be made by analyzing specific scenarios of EET between adjacent IsiA monomers in the ring. Note that for the parameters of the *HexB* model above, the lowest-energy state is well localized on one Chl molecule (even though this molecule can vary between different IsiA monomers due to static energy disorder). Thus, sufficiently fast EET from one IsiA monomer to another is possible only if molecules with proper spectral overlap are simultaneously close enough to each other and feature strong enough dipole–dipole coupling (the inter-monomer EET times were determined in Förster approximation here and ranged from several picoseconds to multiple nanoseconds, sometimes exceeding the radiative lifetime). Furthermore, at every EET step, the excitation energy is somewhat lowered, reducing the probability of further EET within the ring (smaller probability to find available acceptors) and increasing the probability of emission. At the same time, the probability of EET from a given IsiA monomer to the PSI_3_ core is determined by the distance to the relevant acceptors in the core and relevant spectral overlaps. This type of analysis, presented in [64], revealed that there are most likely three entry points for EET from the IsiA_18_ ring to the PSI_3_ core per PSI core monomer, with two entry points located on adjacent IsiA monomers (i.e., nine entry points per PSI_3_ trimer). These data are consistent with the coupling constants between low-energy IsiA 514, 517, 511 Chls, and PSI_3_ core Chls close to the IsiA ring. Possible major entry points from the hexamer to the PSI monomer in model *HexB* are shown in Figure 22, where the yellow arrows indicate likely excitation entry points from the IsiA hexamer to the PSI monomer. Based on the coupling constants, the thicker the arrow, the more likely is the EET from the hexamer to the PSI monomer. As proposed in [64], the major entry point for EET to each the PSI monomer is the B31–B32 dimer, which is one of the PSI_3_ core low-energy traps (C706 trap); see Section 4. Other weaker EET channels were also discussed in [64]. Very recently, a study emerged combining an analysis of cryo-EM data and room-temperature SPCS data for PSI_3_–IsiA_18_ supercomplexes from the same source studied in [64]. In that study, alternative EET pathways from the IsiA_18_ ring to the PSI_3_ core were proposed, involving Chls in PsaK and PsaJ subunits [93] (respective Chs are also shown in Figure 22). However, a calculation of the EET rates in [93] the involved generic spectral overlaps those originally proposed for the LHCII complex [174] and was not necessarily based on the transition energies of the respective Chls that are still a matter of debate.

Three well-separated entry points or more than three entry points per PSI monomer would result in less IsiA_18_ ring emission than shown in Figure 17, while one entry point per PSI core monomer would clearly result in too much IsiA ring emission compared to the experiment. Such an arrangement would render the addition of the IsiA ring antenna nearly useless from a light-harvesting standpoint. The estimated emission of the ring in the presence of three spaced entry points, as shown in Figure 22, is in good agreement with the experiment and it is less red-shifted than the lowest exciton state of a set of six IsiA monomers (i.e., the hexamer), not to mention the whole 18-subunit ring. Integral intensity of the ring emission of around 10% of the total supercomplex emission and a smaller red shift observed in some earlier works [68,158] imply three separate EET entry points, evenly distributed, or more than three entry points per PSI monomer, so there is never more than one inter-IsiA EET step before EET to the PSI core. Thus, the sample studied in [64] appears to have lower ring–core EET probability than those earlier samples. One can speculate that disruption of one entry point and increased absorption of the red region of the PSI core might be related. Indeed, increased coupling between core pigments can cause both the shifts of the core excitonic states and the redistribution of their oscillator strength. A small shift of the core acceptor states/pigments (especially the blue shift that can render potential acceptors useless as opposed to just decreasing the spectral overlaps) could cause significant change of the EET effectiveness through one of the entry points.

Summarizing, various low-temperature spectra reported in [64] for PSI_3_–IsiA_18_ supercomplexes are best described within the so-called *HexB* model, in which the lowest energy pigments of the intact IsiA are assigned to Chl 511 (similar to. CP43′s Chl 44 [54,68]), 514 (similar to. CP43′s Chl 37) [68], and the newly discovered Chl 517. The values of *E*_0_ and *Γ_inh_*, respectively, of Chl 514 and Chl 511 are 14,713 and 153 cm^−1^, as well as 14,717 and 61 cm^−1^, respectively. The E_o_ and fwhm values for Chl 511 (partly lost in isolated monomers) are 14,717 and 61 cm^−1^, respectively. The E_o_ and fwhm values for Chls 508 and 517 (fully missing in isolated monomers) are 14,991 and 106 cm^−1^, as well as 14,770 and 185 cm^−1^, respectively. That is, nearly identical site energies of Chls 514 and 511 and the reversed extent of their inhomogeneous broadening, as compared to previous works [68], provided the best simultaneous fit of multiple spectra, including a reasonable fit of the CD spectra from [158]. Very similar calculated spectra were obtained for all three hexamers, as well as for the entire IsiA_18_ ring. Regarding the isolated monomers, it was shown that model *MB* [64] is also in very good agreement with the experiment, under the assumption that about 2.7 Chls per monomer are absent in the isolated IsiA complexes. This is also consistent with the monomer emission being blue-shifted compared to the 686 nm IsiA_18_ ring emission and sample-dependent maxima of isolated monomer fluorescence spectra (varying from 682–685 nm), as reported in the literature [68,158]. Finally, model *MB* (assuming the absence of Chls 508 and 517 and a 70% loss of Chl 511) provides a better fit to the observed EET dynamics. The lowest-energy Chl 514 (in monomers n, W, and t; see Figure 22) with large inhomogeneous broadening has also the shortest distance between IsiA Chls and PSI red Chls (i.e., B31–B32 dimer, likely the C706 trap [63,66]). Three main energy flows from the IsiA to the PSI_3_ core likely occur via monomer n, as well as monomers W and t of the other two hexamers to the C706 traps (and subsequently to the C714 traps). Additional energy flows occur via monomers r and h (and equivalent ones in other hexamers).

## 6. Concluding Remarks

Frequency-domain studies, including high-resolution optical spectroscopies, remain widely used in the area of photosynthesis research. Above, we presented just a few examples of the frequency-domain spectroscopy data obtained for PSI and PSI supercomplexes, as well as modeling of selected optical spectra. Resent research on the PSI of *Synechocystis* PCC 6803 advanced our understanding of low-energy states in this important photosynthetic complex. High-resolution low-temperature optical spectra obtained for WT PSI and its three mutants (Red_a, Red_b, and Red_ab) shed more light on the energy/composition of the lowest-energy states [63]. Absorption, emission, and resonant and non-resonant NPHB spectra provided a better insight into mutation-induced effects (at the molecular level). By now, it is well established that the lowest-energy state in WT *Synechocystis* PCC 6803 (i.e., C714 trap) is localized on the B37–B38 dimer [63], suggesting very efficient energy migration to P700 RC at physiological temperatures. We demonstrated the presence of two distinct states peaked around 706–707 nm (i.e., degenerate in WT). The C707 state in WT *Synechocystis* PCC 6803 is localized on the B7–A31–A32-His94 cluster [63], which transfers excitation energy to the neighboring lowest energy C714 trap. A mutational study revealed that the C706 state in both the Red_a and Red_ab mutant changed to the C710 state upon transformation of the B31–B32 dimer to the B31–B32–B33 trimer. However, low-temperature emission spectra suggested that EET from C706 to the C714 trap in WT PSI and Red_b mutant is not perfect, as indicated by a weak emission observed near 712 nm. Importantly, a comparison of the optical spectra obtained for Red_ab/Red_b with the Red_a mutant revealed that upon mutation of His94 to Gln, the C707 changes to C716 state in both the Red_b and Red_ab mutants. The resonant HB spectra revealed large el-ph coupling strength that is consistent with a strong mixing of excited states with intermolecular CT states, leading to significantly red-shifted emission spectra. An HB study (as revealed by different shapes and shifts of the blue- and red-shifted antiholes) exposed that both single and double mutations significantly modify the protein energy landscape. 

Comparative studies of the PSI_3_–IsiA_18_ supercomplex, the IsiA ring (IsiA_18_) surrounding the PSI trimer (PSI_3_), and the IsiA monomers revealed that about 2.7 Chls were lost in the isolated monomeric IsiA complexes, at least in the samples studied in [64]. The best fits for isolated monomer spectra [64] were obtained assuming the absence of Chl 508 and Chl 517 and a 70% loss of Chl 511. In turn, the best model describing all three hexamers and the entire IsiA_18_ ring suggested that the lowest energy pigments are Chls 511, 514, and 517. Based on the modeling results, it was concluded that there are most likely three exit points for EET from each IsiA_6_ hexamer to the respective PSI core monomer, with two of these points likely being located next to each other [64]. This corresponds to nine exit/entry points from the IsiA_18_ ring to the PSI_3_ trimer. However, some scenarios with two separated entry points per PSI monomer cannot be entirely excluded. Modeling studies revealed that EET inside individual IsiA monomers is fast (<2 ps at *T* = 5 K), at least 20 times faster than inter-monomer energy transfer [64]. Three well-separated entry points (or more) per PSI monomer could be present in the samples studied by other groups, as suggested by the lower relative intensity of the IsiA emission reported in [7,158]. Frequency-domain studies also suggest the IsiA_18_ ring could act as an energy dissipater (protecting PSI from damage by excessive light exposure [160,161,162]).

It is possible that the bacteria described so far in the photosynthesis literature are not grown under truly identical conditions (e.g., the same amount of light exposure), and as a result, they could develop somewhat different relative arrangements of the IsiA ring, the PSI core, and energy dissipation pathways. The latter could modify emission from the IsiA_18_ ring observed in the literature [62,64,68,158], i.e., a different number of IsiA_18_ → PSI_3_ entry points could be a genuine adaptive feature, given the need to dissipate more energy before it reaches the RC. In any case, the work described in [64] suggested that their PSI supercomplexes likely had nine entry points from the IsiA_18_ to the PSI_3_ trimer, with the best entry point for each PSI monomer likely being the C706 trap assigned in [63] to the B31–B32 dimer (see Figure 22).

## 7. Future Directions

Many excellent papers published over the years, including papers in the current special issue, have provided great insight into the molecular and excitonic structure, as well as dynamics of EET in the PSI core, PSI core mutants [63], and PSI_3_ supercomplexes [62,64,68]. To further improve the quality of the current exciton models, continued efforts are required to generate higher resolution (than currently available [108,109,110,111,112,113,114,115,116,117,118,119,120,121,122,123,124,125,126]) X-ray and cryo-EM structures of these important PCs. In addition, the uncertainties in Chl *Q_y_* site energies and in Chls phonon spectral densities should be decreased to properly simulate both time- and frequency-dependent data obtained at different temperatures. That is, due to the large number of spectrally overlapping Chls in PC, experimental methods often fail to definitively identify the properties of specific individual chromophores. However, first-principles-based modeling protocols promise to predict various properties of pigments in a protein environment to a high precision [50]. Further development of novel computational methods that can be used for predictive modeling of various intact and mutated photosynthetic pigment–protein complexes must continue. 

Regarding information on phonon spectral densities for low-energy pigments in PSI_3_ and PSI_3_–IsiA_18_ supercomplexes, the ∆FLN spectra obtained with red-state excitation will be particularly useful in this regard. Single complex spectroscopy of various mutants of PSI_3_ may provide more insight on distinct red-Chls forms, specifically on their inhomogeneous distributions (HB action spectra that are usually used to visualize the inhomogeneously broadened SDF may not be very useful in this case due to very strong el-ph coupling). Whenever possible, all types of experiments should be performed on samples from the same preparation. It is difficult to simultaneously model multiple spectra if different spectra are obtained in different laboratories, especially when the quality and/or history of the samples cannot be fully assessed. The latter is critical, as loss of some Chls during isolation/purification procedures has been observed. Thus, a reliable set of experimental and simulated data will help to confirm and/or update current energy transfer pathways, in particular, EET to the RC and red traps, in these important PCs. Interestingly, mutant PSI show tendency for aggregation, and somewhat different emission spectra are often reported for the same complexes. Thus, additional care should be taken in analyzing emission spectra. Furthermore, one needs to independently confirm that the C707 state in WT *Synechocystis* PCC 6803 is indeed localized on the B7–A31–A32-His457 cluster, which transfers excitation energy to the neighboring lowest-energy C714 trap (localized on the strongly coupled B37–B38 dimer). Based on many spectroscopic studies, the composition of the C714 trap seems settled. Mutational studies, in turn, revealed that the C706 state in both Red_a and Red_ab mutant changes to the C710 state upon transformation of the B31–B32 dimer to the B31–B32–B33 trimer [63]. It might be of interest to grow an analog of Red_a mutant in iron-stress conditions to determine if the shift of the C706 state to 710 nm is accompanied by any change in effectiveness of the EET from IsiA_18_ to the PSI_3_ core. More research is also needed to reveal true efficiency of the EET from C706 to the C714 trap in both WT PSI and Red_b mutant.

The HB, as well as SPCS data show that high el-ph coupling strength (revealed via resonant HB spectra and single-complex spectra) is consistent with a strong mixing of excited states with intermolecular CT states, leading to significantly red-shifted emission spectra. That the lowest energy state in WT *Synechocystis* PCC 6803 (C714) is localized on the B37–B38 dimer (ensuring efficient energy transfer to P700 at physiological conditions) should be independently confirmed by theoretical modeling studies. It would also be of interest to provide more insight into the energy landscape of WT PSI_3_ and its mutants, i.e., to reveal to what extent single and/or double mutations modify the potential energy landscape in these important complexes, as suggested by different shapes and/or shifts of the blue and red antiholes. The latter can be addressed by exploring both the burning and recovery of resonant holes burned at various frequencies, as well as the recovery of non-resonant holes. Moreover, before modeling of various types of low-temperature optical spectra should continue, shedding more light into the electronic structure of this important photosynthetic complex, more parameters necessary for modeling should be determined experimentally. Parameters of interest include el-ph coupling strength, line shape functions, phonon spectral densities, vibrational frequencies, and inhomogeneous broadening; all these parameters would allow for more reliable modeling of various optical spectra. The information on the location and spectral properties of the red pigments alone is not sufficient to establish the whole picture of energy landscape and EET pathways of the entire PSI_3_ core, or to completely determine how these red Chls control PSI_3_ function.

Somewhat varying emission spectra were observed in the IsiA_18_ ring in the literature [62,64,68,158], but a different number of entry points could be a genuine feature related to adaptation and a need to dissipate more energy before it reaches the PSI RC. This could be studied in future experiments by growing bacteria at different light exposure conditions. Regarding the oxidation state of the P700 state in WT PSI_3_ and in all the PSI_3_ mutants, it should be well controlled to ensure proper interpretation of persistent HB spectra, as P700^+^ may effectively quench the excitations of at least some of the lowest-energy antenna states responsible for fluorescence [67,142]. One could excite PSI_3_ mutants at wavelengths up to 800 nm to test whether mutation changes the EET pathway(s) to the oxidized P700 dimer [67,138,142,170,171,172]. Molecular identity of CT states in PSI RC, as well as in the PSI_3_ core is still an open question; therefore, identification and inclusion of CT state(s) is critical in proper modeling of various optical spectra of PCs. It is widely believed that deciphering the structure–function relationship and electronic structure of the PSI_3_ core, PSI_3_ mutants, and PSI_3_–IsiA_18_ supercomplexes, as well as the nature of red-states in these complexes, will lead to a better understanding of the design rules that drove the evolution of these important complexes [175,176,177,178].

In terms of data analysis, a wide range of machine-learning (ML) techniques may be exploited to establish connection between optical spectroscopy data and the activity of the photosynthetic apparatus of plants and phytoplankton [179]. ML represents a ubiquitous tool that can provide new methods of data analysis [180]. For example, it has been shown recently that ML methods can accelerate the construction of effective Hamiltonians by predicting excited state energies of excitonic states from Coulomb matrices [181]. ML approaches should be applied to PSI core and PSI supercomplexes to bypass computationally costly simulations of open quantum system dynamics in the context of EET [181]. The latter work showed, in the case of FMO, that trained neural networks can reduce computational cost by several orders of magnitude. Their predicted transfer times revealed higher accuracies than frequently used approximate methods, such as secular Redfield theory [182].

We anticipate that explosion of structural data for various PCs and a combination of frequency- and time-domain methodologies (e.g., the ultrafast spectroscopy techniques and coherent 2DES optical spectroscopy) will further advance our ability to track in detail the excitonic structure and the energetic dynamics of various photosynthetic light-harvesting systems.

## Abbreviations

2DES	two-dimensional electronic spectroscopy
B(Chl)	Bacterio(chlorophyll)
Car	carotenoid
CD	circular dichroism
Chl(s)	chlorophyll(s)
Cryo-EM	cryogenic electron microscopy
CW	continuous wave
DFT	density functional theory
∆FLN	difference fluorescence line-narrowing (spectroscopy)
EET	excitation energy transfer
el-ph	electron–phonon (coupling)
ET	electron transfer
FT	Fourier transform
FLN	fluorescence line-narrowing
*Γ_hom_*	homogeneous width
*Γ_inh_*	inhomogeneous width; width of the SDF
Gln	glutamine
HB	(spectral) hole-burning
His	histidine
*J_ph_*(*ω*)	phonon spectral density
*J_vib_*(*ω*)	vibrational spectral density
LH2	light-harvesting complex 2 (of purple bacteria)
LHCII	light-harvesting complex II of green plants
ML	machine learning
NPHB	non-photochemical (spectral) hole burning
P700	primary electron donor of Photosystem I
PC	photosynthetic complex, pigment–protein complex
PSI	Photosystem I
PSI_3_	trimeric cyanobacterial Photosystem I core
PSI_3_–IsiA_18_	PSI–IsiA supercomplex
PSII	Photosystem II
PSB	phonon sideband
RC	reaction center
Red_a mutation	insertion of four amino acids coordinating the additional Chl B33
Red_b mutation	His95 was mutated to Gln95 of *Synechocystis*
Red_ab mutation	both of the above
*S*	el-ph coupling strength (Huang–Rhys factor)
SDF	site distribution function
SPCS	single pigment–protein complex spectroscopy
*Synechocystis*	Cyanobacterium *Synechocystis* (PCC 6803 unless specified otherwise)
*T*	temperature
*T. elongatus*	Cyanobacterium *Thermosynechococcus elongatus*
TLS	two-level system (double-well potential)
WT	wild-type
ZPH	zero-phonon hole
ZPL	zero-phonon line
